# FPFT-2216, a Novel Anti-lymphoma Compound, Induces Simultaneous Degradation of IKZF1/3 and CK1α to Activate p53 and Inhibit NFκB Signaling

**DOI:** 10.1158/2767-9764.CRC-23-0264

**Published:** 2024-02-06

**Authors:** Daiki Kanaoka, Mitsuo Yamada, Hironori Yokoyama, Satoko Nishino, Naoshi Kunimura, Hiroshi Satoyoshi, Shota Wakabayashi, Kazunori Urabe, Takafumi Ishii, Masato Nakanishi

**Affiliations:** 1 Department of Scientific Research, Fujimoto Pharmaceutical Corporation, Nishi-otsuka, Matsubara, Osaka, Japan.

## Abstract

Reducing casein kinase 1α (CK1α) expression inhibits the growth of multiple cancer cell lines, making it a potential therapeutic target for cancer. Herein, we evaluated the antitumor activity of FPFT-2216—a novel low molecular weight compound—in lymphoid tumors and elucidated its molecular mechanism of action. In addition, we determined whether targeting CK1α with FPFT-2216 is useful for treating hematopoietic malignancies. FPFT-2216 strongly degraded CK1α and IKAROS family zinc finger 1/3 (IKZF1/3) via proteasomal degradation. FPFT-2216 exhibited stronger inhibitory effects on human lymphoma cell proliferation than known thalidomide derivatives and induced upregulation of p53 and its transcriptional targets, namely, p21 and MDM2. Combining FPFT-2216 with an MDM2 inhibitor exhibited synergistic antiproliferative activity and induced rapid tumor regression in immunodeficient mice subcutaneously transplanted with a human lymphoma cell line. Nearly all tumors in mice disappeared after 10 days; this was continuously observed in 5 of 7 mice up to 24 days after the final FPFT-2216 administration. FPFT-2216 also enhanced the antitumor activity of rituximab and showed antitumor activity in a patient-derived diffuse large B-cell lymphoma xenograft model. Furthermore, FPFT-2216 decreased the activity of the CARD11/BCL10/MALT1 (CBM) complex and inhibited IκBα and NFκB phosphorylation. These effects were mediated through CK1α degradation and were stronger than those of known IKZF1/3 degraders. In conclusion, FPFT-2216 inhibits tumor growth by activating the p53 signaling pathway and inhibiting the CBM complex/NFκB pathway via CK1α degradation. Therefore, FPFT-2216 may represent an effective therapeutic agent for hematopoietic malignancies, such as lymphoma.

**Significance:**

We found potential vulnerability to CK1α degradation in certain lymphoma cells refractory to IKZF1/3 degraders. Targeting CK1α with FPFT-2216 could inhibit the growth of these cells by activating p53 signaling. Our study demonstrates the potential therapeutic application of CK1α degraders, such as FPFT-2216, for treating lymphoma.

## Introduction

Thalidomide comprises glutarimide and phthalimide and is effective against multiple myeloma, erythema nodosum leprosum, and POEMS (polyneuropathy, organomegaly, endocrinopathy, M-protein, and skin changes) syndrome (1–3). Specific thalidomide derivatives, namely lenalidomide and pomalidomide, are also therapeutic agents for multiple myeloma (4, 5). In addition, next-generation thalidomide derivatives, including avadomide, iberdomide, CC-90009 (eragidomide), CC-92480 (mezigdomide), and CC-99282 (golcadomide), are currently under development for hematopoietic malignancies (4, 5). Thalidomide and its derivatives bind to cereblon (CRBN)—a substrate receptor for ubiquitin E3 ligases—via glutarimide (1, 2, 4–6) and recruit proteins, such as IKAROS family zinc finger 1/3 [i.e., Ikaros/Aiolos (A/I)], casein kinase 1α (CK1α), phosphodiesterase 6D (PDE6D), and G_1_- to S-phase transition 1 (GSPT1), to CRBN as neosubstrates (7–11). Thalidomide derivatives exhibit tumor growth inhibition and immunomodulatory activity due to CRBN neosubstrate ubiquitination and proteasomal degradation (4, 5). The differences in the phthalimide groups of thalidomide derivatives confer substrate selectivity, contributing to their diverse pharmacologic actions.

CK1α is a serine/threonine kinase encoded by *CSNK1A1* on chromosome 5q32 with an oncogenic role in certain cancers (12–14). CK1α inhibits the function of the tumor suppressor p53 (15, 16), while CK1α inhibition or silencing activates the p53 signaling pathway (15–18). Furthermore, in diffuse large B-cell lymphoma (DLBCL) and mantle cell lymphoma (MCL), CK1α contributes to the constitutive activation of the NFκB pathway along with the CARD11/BCL10/MALT1 (CBM) complex (19–21). Notably, CK1α knockdown inhibits the NFκB pathway (19). Hence, inhibition or decreased CK1α expression induces growth inhibition in various cancer cell lines (15–21), suggesting that it may serve as a promising therapeutic target for cancer.

Unlike A/I degraders, including thalidomide and pomalidomide, lenalidomide has a CK1α-degrading effect (5, 9) and has been used to treat several hematopoietic malignancies, including DLBCL, follicular lymphoma (FL), MCL, and 5q deletion myelodysplastic syndrome [del(5q) MDS] (4, 5, 9). The CK1α-degrading effect is partially responsible for the therapeutic effect of lenalidomide; however, its significance remains unclear (12, 22). During our screening for novel thalidomide derivatives with potent immunomodulatory and antitumor activity, we identified FPFT-2216 as a promising compound that possesses a novel scaffold comprising a triazole and thiophene ring that is absent from known thalidomide derivatives (23). Compared with lenalidomide, FPFT-2216 exhibits potent degradation activity against CK1α and A/I (11, 23) and serves as an effective chemical probe to clarify the roles of CK1α in hematopoietic malignancies.

In this study, we evaluated the *in vitro* antitumor activity of FPFT-2216 in lymphoid tumors and elucidated its molecular mechanism of action. Moreover, we assessed whether FPFT-2216 exhibits *in vivo* antitumor effects and can serve as a therapeutic agent for hematopoietic malignancies.

## Materials and Methods

### Reagents

FPFT-2216 and pomalidomide were synthesized by Fujimoto Pharmaceutical Group. Avadomide, iberdomide, siremadlin, idasanutlin, navtemadlin, milademetan, and MG-132 were procured from MedChemExpress; Z-VRPR-FMK and phorbol 12-myristate 13-acetate (PMA) were procured from Adipogen Life Sciences. Lenalidomide and ionomycin were obtained from Cayman Chemical, BAY 11-7082 from FUJIFILM Wako Pure Chemical Corporation, and safimaltib from Selleck Chemicals. Rituxan Intravenous Infusion (rituximab) was obtained from Zenyaku Kogyo. Z-VRPR-FMK was dissolved in PBS, and other reagents were dissolved in DMSO. The final DMSO concentration used for *in vitro* experiments did not exceed 0.25%.

### Cell Culture

*Mycoplasma*-free human hematopoietic tumor cell lines and human embryonic kidney-derived HEK293T cells were obtained from ATCC, DSMZ, Japanese Collection of Research Bioresources, and Horizon Discovery between 2009 and 2021 (Supplementary Table S1 for details). Whole blood samples were collected from healthy volunteers at the Fujimoto Pharmaceutical Group on-site blood donation unit. All blood samples were obtained with appropriate consent and in accordance with the research ethics and compliance program of Fujimoto Pharmaceutical Group. Human peripheral blood mononuclear cells (PBMC) were isolated from the whole blood using Lymphoprep (Axis-Shield). The cells were cultured at 37°C under 5% CO_2_ in RPMI1640 or DMEM (Nacalai Tesque) supplemented with 100 units/mL of penicillin (Thermo Fisher Scientific), 100 µg/mL streptomycin (Thermo Fisher Scientific), and 10% or 20% FBS (Thermo Fisher Scientific). Cellular authentication was performed through short tandem repeat analysis at the source of purchase. After acquisition, cells were tested for *Mycoplasma* infection using the MycoAlert Mycoplasma Detection Kit or MycoAlert PLUS Mycoplasma Detection Kit (Lonza; latest run date: November 2023) and were negative. A portion of the cells were tested for *Mycoplasma* infection at the source of purchase. Cells were used within 20 passages after thawing in all experiments.

### Cell Growth Inhibition Test

To evaluate the antiproliferative activity of FPFT-2216, lymphoid tumor cell lines treated with FPFT-2216, thalidomide derivatives, or safimaltib were seeded in 96-well plates and cultured at 37°C under 5% CO_2_ for 3 days. In a combination study, cells were cotreated with FPFT-2216 and an MDM2 inhibitor. PBMCs were seeded on an anti-CD3 antibody-immobilized (BioLegend, catalog no. 317326, RRID: AB_11150592) 96-well plate and treated with FPFT-2216 or thalidomide derivatives. A WST-8 kit (Kishida Chemical Laboratory) was used according to the manufacturer's instructions, and absorbance was measured using a SpectraMax M5 (Molecular Devices, RRID: SCR_020300) to evaluate cell viability. The percentage of absorbance in the compound-treated cells was calculated relative to the absorbance in DMSO-treated cells (control), designated as 100%, and defined as the cell viability (%). The IC_50_ value in Table 1 was calculated with Graph Pad Prism 5.04 (GraphPad Software, RRID: SCR_002798) using the cell viability results of three independent experiments.

**TABLE 1:** IC_50_ values of FPFT-2216 and thalidomide derivatives against lymphoid tumor cell lines

				IC_50_ (µmol/L)				
Tumor type	Cell line	p53 status^a^	MALT1i sensitivity^b^	2216	Lena	Poma	Avad	Iber
DLBCL (GCB)	Pfeiffer	Mutant	Unknown	>100	>100	>100	>100	>100
	SU-DHL-4	Mutant	Resistant	>100	>100	>100	>100	N. D.
	RC-K8	Wild-type	Resistant	32.839	>100	N. D.	N. D.	29.587
	WSU-DLCL2	Mutant	Unknown	16.064	>100	>100	1.375	>100
DLBCL (non-GCB)	SU-DHL-2	Mutant	Resistant	>100	>100	>100	>100	N. D.
	U-2932	Mutant	Resistant	45.690	>100	>100	>100	>100
	RI-1 (RIVA)	Mutant	Sensitive	0.603	>100	>100	>100	>100
	OCI-Ly3	Wild-type	Sensitive	0.090	>100	N. D.	N. D.	17.501
MCL	MAVER-1	Mutant	Resistant	>100	>100	>100	>100	>100
	JeKo-1	Mutant	Sensitive	>100	>100	>100	>100	>100
	GRANTA-519	Wild-type	Resistant	>10	>10	N. D.	N. D.	N. D.
	JVM-2	Wild-type	Resistant	>10	>10	N. D.	N. D.	N. D.
	MINO	Mutant	Sensitive	0.226	1.025	N. D.	N. D.	N. D.
	Z-138	Wild-type	Resistant	0.140	>100	>100	>100	>100
	REC-1	Mutant	Sensitive	0.012	0.200	0.031	0.040	0.004
FL	Minami-1	Unknown	Unknown	>100	>100	>100	>100	>100
	RL	Mutant	Unknown	3.466	>10	N. D.	N. D.	N. D.
	DOHH-2	Wild-type	Unknown	0.465	>100	>100	1.561	1.084
BL	CA46	Mutant	Unknown	>100	>100	>100	>100	>100
	Ramos (RA1)	Mutant	Unknown	3.312	>100	>100	24.983	1.853
	Daudi	Mutant	Unknown	0.038	2.841	0.106	0.097	0.006
ALL	SEM	Mutant	Unknown	>100	>100	>100	>100	>100
	NALM-6	Wild-type	Unknown	>100	>100	>100	>100	>100
	Kasumi-8	Wild-type	Unknown	>100	>100	>100	>100	>100
	KHM-2B	Unknown	Unknown	23.430	>100	>100	4.005	>100
	REH	Mutant	Unknown	7.080	>100	>100	>100	>100
	RS4;11	Wild-type	Resistant	0.351	>100	>100	>100	>100
	Kasumi-7	Wild-type	Unknown	0.334	>100	>100	2.490	1.430
	Kasumi-10	Wild-type	Unknown	0.093	>100	>100	>100	>100
Normal cell	PBMCs	Unknown	Unknown	>100	>100	>100	N. D.	>100

Abbreviations: 2216, FPFT-2216; Lena, lenalidomide; Poma, pomalidomide; Avad, avadomide; Iber, iberdomide; N. D., not determined.

^a^The p53 status was based on the TP53 database (https://tp53.isb-cgc.org/), Cellosaurus (https://www.cellosaurus.org/), and several references (29–33, 43, 44).

^b^MALT1i sensitivity indicates the sensitivity of cell lines to growth inhibition by Z-VRPR-FMK (MALT1 caspase inhibitor peptide) or other compounds and was based on several references (38, 40, 50) and Supplementary Table S4.

### Ectopic CK1α G40N Mutant Expression

To clarify the involvement of CK1α degradation on FPFT-2216 activities, we used the pLV-SFFV Pur vector, which replaced the promoter with spleen focus-forming virus (SFFV), for CK1α mutant expression experiments. The CK1α G40N mutant, in which the glycine at position 40 encoded by *CSNK1A1* (accession no.: NM_001892.6) was replaced with asparagine, was cloned into the pLV-SFFV Pur vector. HEK293T cells were cotransfected with the CK1α G40N expression plasmid vector and 2nd Generation Packaging System Mix (ABM) using PEI MAX (Polysciences, Inc.). Virus-containing culture supernatants were harvested after 3 days. Lymphoid tumor cell lines were infected with a virus in the presence of polybrene in the growth medium. During this period, cells transduced with a GFP-expressing empty vector were used as a control for CK1α mutant expression experiments. A stable expression strain was obtained by culturing the virus-infected cell line in the presence of puromycin (InvivoGen).

### Western Blot Analysis

Briefly, cells treated with FPFT-2216, thalidomide derivatives, or Z-VRPR-FMK were lysed using Halt Protease and Phosphatase Inhibitor Single-Use Cocktail-containing RIPA buffer (Thermo Fisher Scientific) to analyze the molecular mechanism of FPFT-2216 action. Cells were cotreated with FPFT-2216 or thalidomide derivatives and MG-132 or siremadlin in some experiments. Ultrasonic treatment was performed according to the instructions, and centrifugation was performed at 10,000 × *g* for 10 minutes at 4°C. The protein concentration of the extract was measured using the DC Protein Assay (Bio-Rad) or Pierce 660 nm Protein Assay Kit (Thermo Fisher Scientific). SDS-PAGE samples were prepared using Sample Buffer Solution with Reducing Reagent (6×; Nacalai Tesque). The samples were subjected to electrophoresis (7.5–100 µg protein) using polyacrylamide gel (ATTO Corporation or Nacalai Tesque) at the optimum concentration (10%, 7.5%–15%, or 5%–20%) and transferred onto a polyvinylidene difluoride (PVDF) membrane (ATTO Corporation or Merck Millipore). The PVDF membrane was blocked with 5% skimmed milk solution in TBS containing 0.05% Tween 20 (TBST), Bullet Blocking One (Nacalai Tesque), or Blocking One-P (Nacalai Tesque). The blots were then incubated with the primary antibody overnight at 4°C (Supplementary Table S2). Signal Enhancer HIKARI (Nacalai Tesque) was used as an antibody reaction solution to detect phosphorylated IκBα. After washing the primary antibody with TBST, the PVDF membrane was incubated with horseradish peroxidase (HRP)-conjugated secondary antibodies (goat anti-rabbit, Abcam, catalog no. ab7090, RRID: AB_955417 and goat anti-mouse, Abcam, catalog no. ab97040, RRID: AB_10698223) at room temperature (22°C ± 5°C) for 1 hour. Secondary antibodies were removed using TBST, and immunoreactive signals were detected with Immobilon Western HRP substrate (Merck Millipore). In some experiments, WB Stripping Solution Strong (Nacalai Tesque) was used to strip the membranes before reblotting to detect additional proteins. Band intensity was quantified using Image Lab analysis software version 3.0 (Bio-Rad, RRID: SCR_014210).

### ELISA

To determine FPFT-2216 activity in immunomodulation, Jurkat cells and PBMCs treated with FPFT-2216 or thalidomide derivatives were plated on BioCoat Anti-Human CD3 T-cell Activation Plates (Corning) and cultured at 37°C under 5% CO_2_. After 48 hours, IL2 levels in the culture supernatant were measured using the Human IL2 Standard ELISA Development Kit (PeproTech). The relative IL2 production rate (%) upon treatment with each compound was determined by setting the amount of IL2 produced by DMSO-treated cells to 100%.

### *In Vivo* Cell Line–derived Xenograft Study

All animal experiments were approved by the Animal Care and Use Committee of Fujimoto Pharmaceutical Co., Ltd. (approval numbers AC-F-3157 and AC-F-3184). Five- to 6-week-old male CB17/Icr-*Prkdc^scid^*/CrlCrlj (C.B-17 SCID) mice (17–25 g; The Jackson Laboratory) were bred aseptically. To evaluate the antitumor activity of FPFT-2216, a cell suspension containing 50% Matrigel Basement Membrane Matrix High Concentration (Corning) was injected into the right flanks at a volume of 0.1 mL (Z-138: 1 × 10^7^ cells/mouse; DOHH-2: 0.5 × 10^7^ cells/mouse). Body weight and tumor size were measured two to three times per week. Tumor volume was calculated using the formula: length × width × width × 0.5. Once the tumor volume reached 150 mm^3^ (Z-138 cells) or 200 mm^3^ (DOHH-2 cells), mice were randomly grouped such that the average tumor volume was consistent between groups, and compound administration was initiated (day 1). FPFT-2216 was administered orally once daily for 5 consecutive days per week for 3 weeks in the siremadlin combination study and for 4 weeks in the rituximab combination study. Siremadlin was administered orally twice weekly for 3 weeks. Rituximab was administered as a single intraperitoneal injection on day 1. A follow-up study was performed to determine tumor regrowth after the final FPFT-2216 treatment when tumor regression was observed in a combination group. Tumor volume measurement in each group continued until the tumor volume exceeded 2,000 mm^3^, at which point, the animals were euthanized. A 1% carboxymethylcellulose sodium salt solution was used as the vehicle for FPFT-2216, and 0.5% methylcellulose #400/50 mmol/L phosphate buffer (pH 6.8) was used for siremadlin. Rituximab was diluted with saline.

For the pharmacodynamic study, mice bearing Z-138 tumor xenografts were administered FPFT-2216 once daily for 4 days, and tumors were harvested 6 hours after the final administration. Tumors were homogenized in protease/phosphatase inhibitor–containing RIPA buffer, and lysates were subjected to sonication, protein quantification, and Western blot analysis as described previously.

### *In Vitro/In Vivo* Patient-derived Xenograft Study

To evaluate the anti-lymphoma activity of FPFT-2216 in patient tumors refractory to rituximab-based standard therapy, we used six non-germinal center B-cell (non-GCB) DLBCL patient-derived xenograft (PDX) models (Supplementary Table S3 for details). This PDX study was outsourced to Charles River Laboratories Discovery Research Services and performed according to their protocol. Written informed consent was obtained from all patients prior to tumor donation for the institutional-initiated research studies. All experiments were conducted in accordance with the guidelines of the Declaration of Helsinki and good clinical practice and approved by the ethical commission of the Albert Ludwig University Freiburg (permit-# EK Freiburg: 279/10, 07.09.2010). PDX tumors were grown subcutaneously in female NOD.Cg-*Prkdc^scid^ Il2rg^tm1WjI^*/SzJ (NSG) mice (The Jackson Laboratory, RRID: IMSR_JAX:005557). Subcutaneous PDX tumors were mechanically dissociated into single-cell suspensions and PDX cells were cultured for short-term before FPFT-2216 treatment. PDX cells were treated with FPFT-2216 and cultured at 37°C under 5% CO_2_ for 3 days. The Cell Titer-Glo Luminescent Cell Viability Assay (Promega) was used according to the manufacturer's instructions, and luminescence was measured using an Envision Multimode plate reader (PerkinElmer) to determine cell viability (%).

Four to 6 weeks old female NSG mice were subcutaneously transplanted with LYXFDLBC 2835 tumor cells from a patient with non-GCB DLBCL. Body weight and tumor size were measured two to three times per week. Eleven days after transplantation, the mice were randomly divided into groups, ensuring that the average tumor volume was the same among groups (day 0). FPFT-2216 was then orally administered once daily beginning on day 1 for up to 3 weeks. As a positive control, cyclophosphamide (CPA) was intraperitoneally administered once every 2 weeks for up to 3 weeks until the mice were euthanized. Saline was used as the vehicle for CPA.

### Statistical Analyses

SPSS 23.0 (IBM Corporation; RRID: SCR_002865) was used for statistical analysis. *In vivo,* antitumor activity was evaluated by calculating the test over control (T/C, %) and time to endpoint (TTE, days) from the tumor volume (Table 2). The differences in tumor volume between the vehicle and compound groups and between the single-compound administration and combination administration groups were analyzed using Dunnett or Tukey tests. A two-tailed log-rank test was performed on the median TTE. A *P*-value ≤ 0.05 was considered significant for all tests. The number of animals used in each experiment is indicated in the corresponding figure legends.

**TABLE 2:**
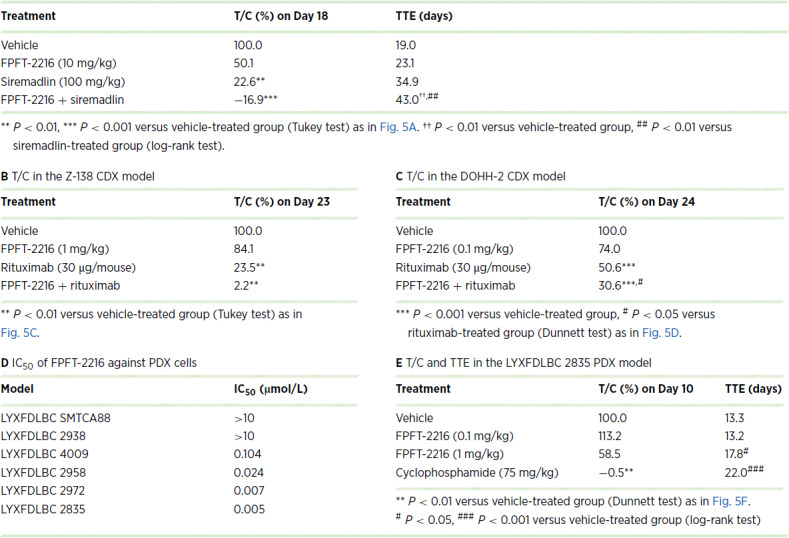
Antitumor activity of FPFT-2216 in cell line–derived xenograft (CDX) and PDX models **A** T/C and TTE in the Z-138 CDX model

NOTE: T/C (%) indicates the percentage of tumor volume change (tumor volume on the day of a measurement minus tumor volume on the day of first administration) in each compound-administered group, with the tumor volume change in the vehicle-administered group as 100%. TTE indicates the number of days from the first administration of the compound (Day 1) until the tumor volume reaches 2000 mm^3^. TTE was calculated as the number of days to reach a tumor volume of 2000 mm^3^ using the linear regression line of logarithmically transformed tumor volume measurements at each time point. If tumor volume was maintained at <2000 mm^3^ until the end of the study, TTE was designated as 22 days (E) or 43 days (A).

### Data Availability Statement

The data supporting the findings of this study are available upon reasonable request from the corresponding author.

## Results

### FPFT-2216 Suppresses Lymphoid Tumor Cell Line Proliferation Through CK1α Degradation

**FIGURE 1:**
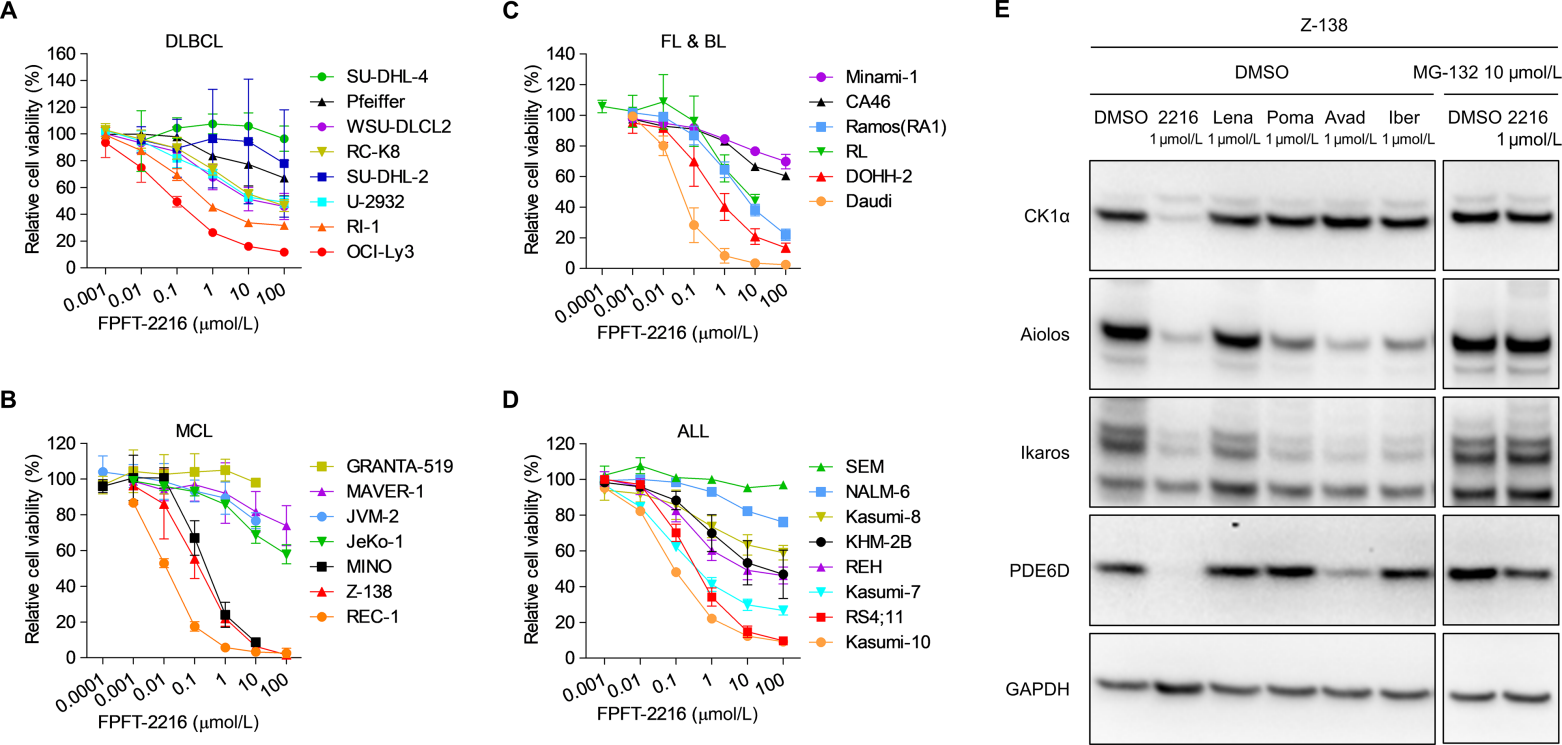
Antiproliferative activity of FPFT-2216 in lymphoid tumor cell lines. **A–D,** Cell viability (%) of DLBCL, MCL, FL, BL, and ALL cell lines cultured for three days in FPFT-2216. Results are presented as the mean ± SD (*n* = 3). **E,** Western blot analysis of Z-138 cells cultured for 6 hours in the presence of different compounds at the concentrations shown in the figure. Representative results from two independent experiments are shown. GAPDH was used as a loading control. 2216, FPFT-2216; Lena, lenalidomide; Poma, pomalidomide; Avad, avadomide; Iber, iberdomide.

We first evaluated the antiproliferative activity of FPFT-2216 in lymphoid tumor cell lines. FPFT-2216 largely suppressed DLBCL cell line proliferation in a concentration-dependent manner and reduced the cell viability to ≤ 50% in five of eight cell lines (Fig. 1A; Table 1). FPFT-2216 suppressed non-GCB DLBCL proliferation more robustly than GCB DLBCL proliferation (Fig. 1A; Table 1). FPFT-2216 also exhibited growth-inhibitory activity against cell lines derived from MCL, FL, Burkitt lymphoma, and acute lymphoblastic leukemia (ALL; Fig. 1B–D; Table 1). The IC_50_ values of FPFT-2216 were 0.090 µmol/L for OCI-Ly3, 0.140 µmol/L for Z-138, 0.351 µmol/L for RS4;11, and 0.093 µmol/L for Kasumi-10 cells (Table 1). In contrast, those of lenalidomide, pomalidomide, avadomide, and iberdomide exceeded 10 µmol/L (Table 1). However, FPFT-2216 did not inhibit PBMC proliferation or survival stimulated with an anti-CD3 antibody (Table 1). These results demonstrate that FPFT-2216 has growth-inhibitory activity against lymphoid tumor cells and exhibits stronger activity than thalidomide derivatives in certain cell lines.

Z-138 cells, the proliferation of which was suppressed by FPFT-2216, were used to investigate the CRBN neosubstrate protein level after treatment with FPFT-2216 and thalidomide derivatives. FPFT-2216 markedly reduced CK1α, A/I, and PDE6D protein abundance; however, these effects were abolished by the proteasome inhibitor MG-132 (Fig. 1E). FPFT-2216 exhibited stronger CK1α-degrading activity than thalidomide derivatives (Fig. 1E). Consistent with a previously reported proteomic analysis (24), avadomide reduced PDE6D protein abundance; therefore, the PDE6D-degrading and growth-inhibitory activities in Z-138 cells were not correlated (Fig. 1E; Table 1).

**FIGURE 2:**
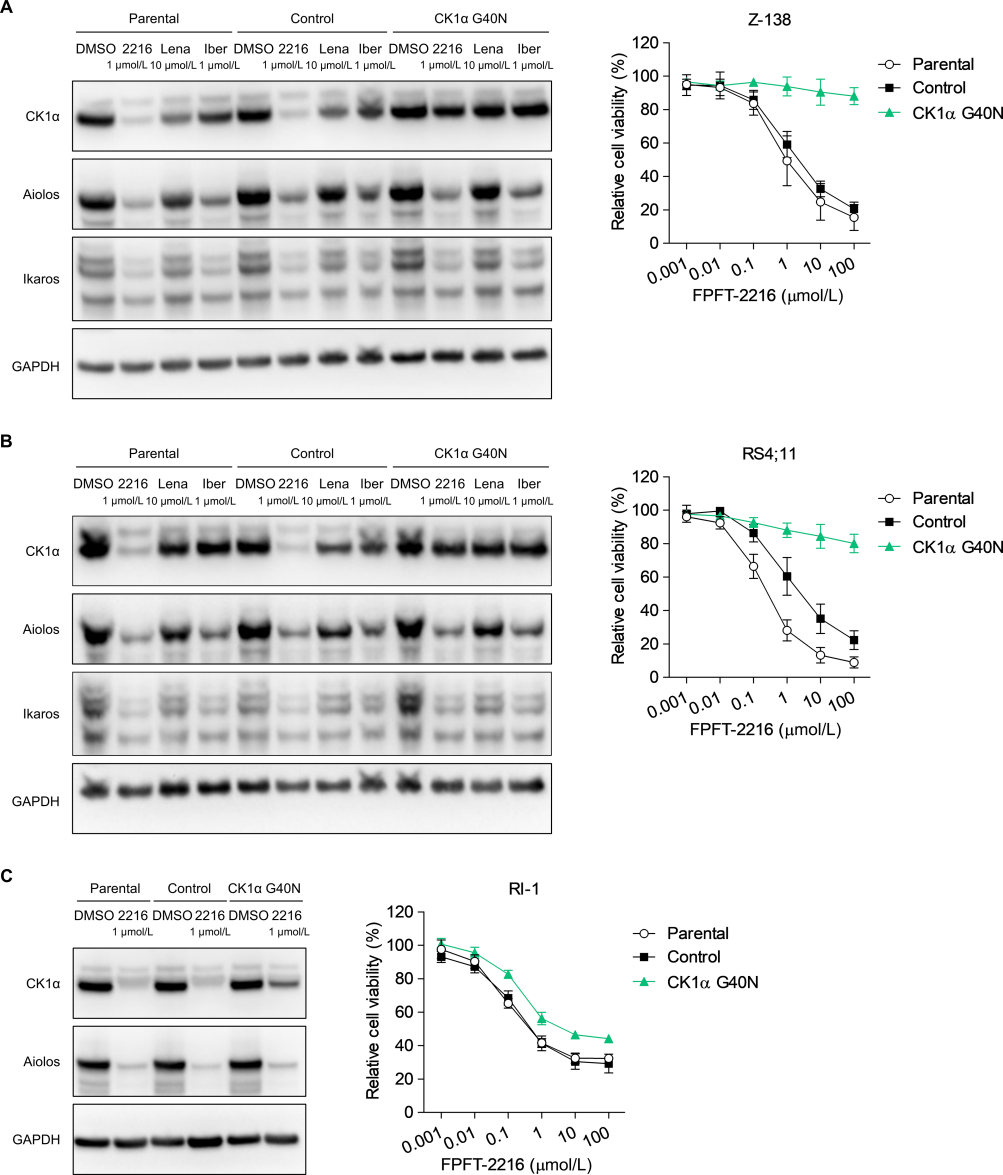
Antiproliferative activity of FPFT-2216 via CK1α degradation. FPFT-2216 degrading activity and growth inhibition in Z-138 (**A**), RS4;11 (**B**), and RI-1 (**C**) cells with a CK1α G40N mutant. Left: Western blot analysis of cells cultured for 6 hours (A and B) or 24 hours (C) in the presence of compounds at different concentrations. Representative results from two independent experiments are shown. GAPDH was used as a loading control. Right: Viability (%) of each cell type cultured for three days in the presence of 0.001–100 µmol/L FPFT-2216. Results are presented as the mean ± SD (*n* = 3). 2216, FPFT-2216; Lena, lenalidomide; Iber, iberdomide.

FPFT-2216 exerted stronger growth-inhibitory and CK1α-degrading activities against lymphoid tumor cell lines than thalidomide derivatives (Fig. 1; Table 1). Therefore, we investigated the involvement of CK1α degradation in the growth-inhibitory activity of FPFT-2216 using the CK1α G40N mutant, resistant to protein degradation by lenalidomide (25–28). In the parental and control Z-138 cells, FPFT-2216 and lenalidomide induced CK1α degradation (Fig. 2A, left). However, these effects were relatively abolished in Z-138 cells expressing the CK1α G40N mutant (Fig. 2A, left). Moreover, FPFT-2216, lenalidomide, and iberdomide enhanced A/I degradation regardless of the CK1α G40N mutant (Fig. 2A, left). Unlike the parental and control, the growth-inhibitory activity of FPFT-2216 was not observed in Z-138 cells expressing CK1α G40N mutant (Fig. 2A, right). The same results were obtained in RS4;11 cells (Fig. 2B). In the case of RI-1 cells, CK1α G40N mutant expression was lower than in Z-138 cells, and its counter effect against the growth-inhibitory activity of FPFT-2216 was incomplete (Fig. 2C). Overall, these results demonstrate that FPFT-2216 suppresses the proliferation of lymphoid tumor cell lines through CK1α degradation.

### FPFT-2216 Exerts Antiproliferative Activity by Activating the p53 Signaling Pathway via CK1α Degradation

**FIGURE 3:**
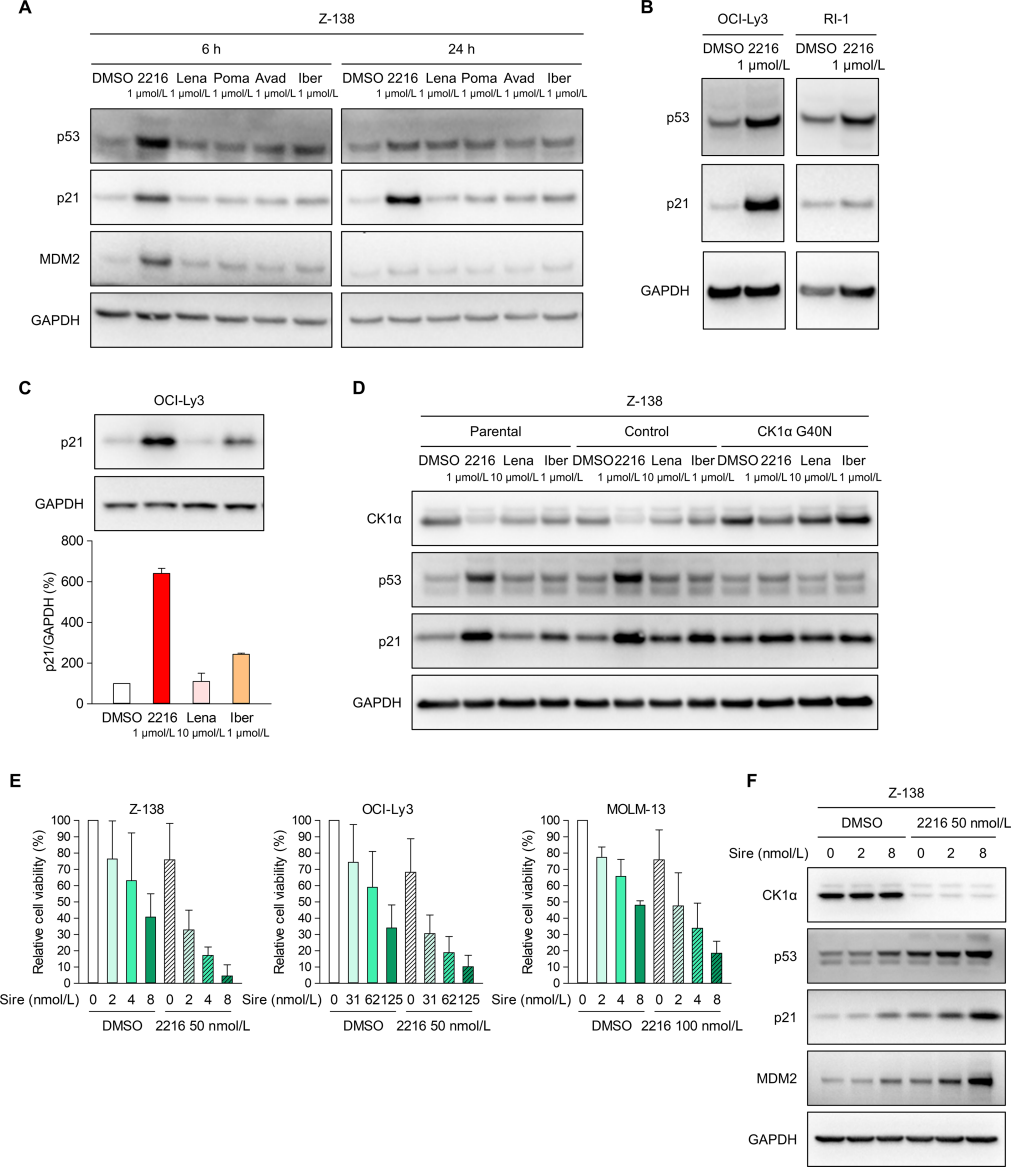
Activation of the p53 signaling pathway via CK1α degradation by FPFT-2216. Western blot analysis of Z-138 cells cultured for 6 and 24 hours (**A**), OCI-Ly3 and RI-1 cells cultured for 24 hours (**B** and **C**), and CK1α G40N mutant-expressing Z-138 cells cultured for 6 hours (**D**) in the presence of compounds at different concentrations. Representative results from two (A and D) or three (B) independent experiments are shown. GAPDH was used as a loading control. In the case of OCI-Ly3 cells, the p21 band intensity was normalized to GAPDH, and the p21/GAPDH band intensity ratio (%) after treatment with various compounds was determined with the p21/GAPDH band intensity ratio in DMSO-treated cells taken as 100% (C, mean ± SD, *n* = 3). **E,** Cell viability (%) of Z-138, OCI-Ly3, and MOLM-13 cells cultured for three days in the presence of FPFT-2216 and siremadlin at the concentrations shown in the figure. Results are presented as the mean ± SD (*n* = 3). **F,** Western blot analysis of Z-138 cells treated for 6 hours with varying concentrations of siremadlin alone or in combination with 50 nmol/L FPFT-2216. Representative results from two independent experiments are shown. 2216, FPFT-2216; Lena, lenalidomide; Poma, pomalidomide; Avad, avadomide; Iber, iberdomide; Sire, siremadlin.

CK1α negatively regulates the transcriptional activity of the tumor suppressor p53 (12, 15, 16). Therefore, FPFT-2216 may exert antiproliferative activity against lymphoid tumor cell lines by activating the p53 signaling pathway through CK1α degradation. Marked increases in p53 protein and the cell cycle arrest factor p21 protein (a transcriptional target of p53) were observed in Z-138, OCI-Ly3, and RS4;11 cells (p53 wild-type cells; refs. 29–31) treated with FPFT-2216 for 6 or 24 hours (Fig. 3A–C; Supplementary Fig. S1A). In Z-S138 cells, p21 upregulation was more pronounced 24 hours after FPFT-2216 treatment than 6 hours (Fig. 3A). Meanwhile, compared with FPFT-2216, known thalidomide derivatives had minor effects on p53/p21 expression (Fig. 3A and 3C; Supplementary Fig. S1A). Mutant p53-expressing RI-1 and p53 wild-type RC-K8 cells (32, 33) showed no significant increase in p53/p21 expression upon FPFT-2216 treatment (Fig. 3B; Supplementary Fig. S1B). Next, we examined p53/p21 in Z-138 and RS4;11 cells expressing the CK1α G40N mutant. As expected, the increase in p53/p21 proteins was relatively abolished with the attenuation of CK1α degradation by FPFT-2216 (Fig. 3D; Supplementary Fig. S1A). These findings indicate that FPFT-2216 induces p53 activation and subsequent p21 upregulation through CK1α degradation in certain lymphoid tumor cell lines.

The expression of the p53 repressor MDM2 increased in Z-138 cells treated with FPFT-2216 (Fig. 3A). When Z-138 cells were treated with FPFT-2216 combined with the MDM2 inhibitor, siremadlin, a stronger antiproliferative activity was observed than when either treatment was used alone (Fig. 3E, left). The enhanced effect of FPFT-2216 on the antiproliferative activity of siremadlin was also observed in OCI-Ly3 and RS4;11 cells (Fig. 3E, middle; Supplementary Fig. S1C). In Z-138 cells, siremadlin stabilized p53 expression and upregulated p21/MDM2; these effects were enhanced by FPFT-2216 (Fig. 3F).

Several MDM2 inhibitors are in clinical trials for acute myeloid leukemia (AML) treatment (34). Therefore, we also evaluated the combined effect of both compounds in the p53 wild-type AML cell line MOLM-13 (35). FPFT-2216 enhanced the antiproliferative action of siremadlin (Fig. 3E, right) and other MDM2 inhibitors (idasanutlin, navtemadlin, and milademetan; Supplementary Fig. S1D and S1E). In RI-1 cells (p53 mutant), siremadlin did not inhibit cell growth, and FPFT-2216 did not enhance the effect of siremadlin (Supplementary Fig. S1F). In summary, FPFT-2216 enhances the antiproliferative activity of MDM2 inhibitors by activating p53 signaling in lymphoid tumors and AML cell lines.

### FPFT-2216 Suppresses CBM Complex Activity/NFκB Pathway via CK1α Degradation

**FIGURE 4:**
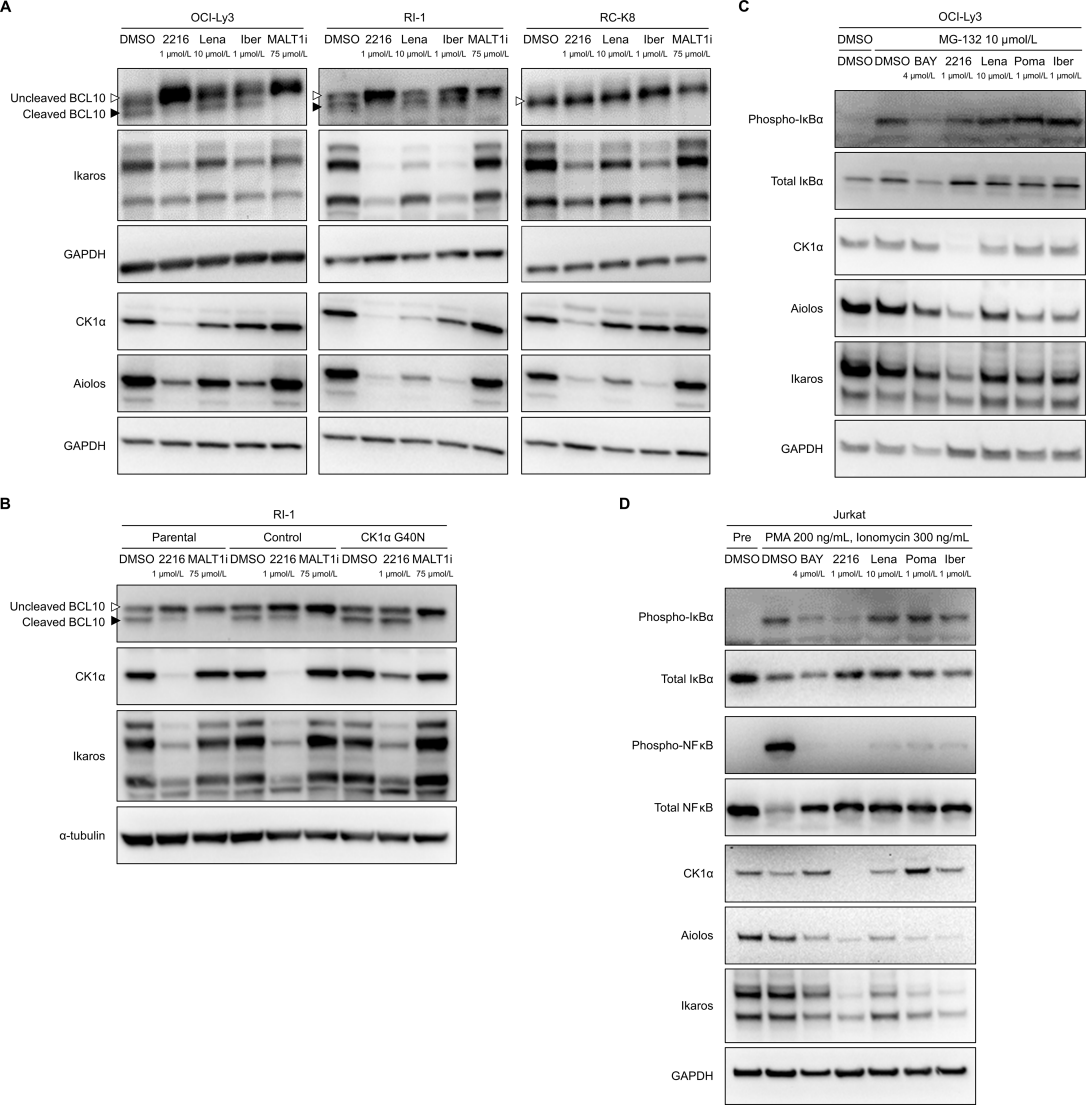
Suppression of CBM complex activity/NFκB pathway via CK1α degradation by FPFT-2216. Western blot analysis of DLBCL cell lines (**A**) and RI-1 cells expressing CK1α G40N mutant (**B**) cultured for 24 hours in the presence of compounds at different concentrations. Open triangle: Uncleaved BCL10, closed triangle: Cleaved BCL10. **C,** MG-132 was added after OCI-Ly3 cells were cultured for 23 hours in the presence of compounds at the concentrations shown in the figure. After 1 hour, proteins were extracted from the cells, and Western blot analysis was performed. **D,** Jurkat cells were cultured for approximately 24 hours in the presence of compounds at the concentrations shown in the figure, and the cells were cultured for 15 minutes after adding PMA/ionomycin. Proteins were extracted from the cells, and Western blot analysis was performed. Representative results from two (B–D) or three (A) independent experiments are shown. GAPDH or α-tubulin was used as a loading control. 2216, FPFT-2216; Lena, lenalidomide; Iber, iberdomide; MALT1i, Z-VRPR-FMK; BAY, BAY 11-7082; Poma, pomalidomide.

FPFT-2216 exhibited stronger inhibitory effects on RI-1 cell (p53 mutant) proliferation than known thalidomide derivatives (Fig. 1A; Table 1), suggesting a mechanism of action other than p53 activation by CK1α degradation. Constitutive activation of the NFκB pathway plays an important role in the proliferation/survival of activated B cell (ABC, currently non-GCB) DLBCL (36), in which the contribution of the CK1α and CBM complex has been reported (19, 20, 37). Notably, lenalidomide inhibits MALT1-mediated BCL10 cleavage (an indicator of CBM complex activation) and suppresses NFκB activity in non-GCB DLBCL cell lines (22). Therefore, we investigated the effect of FPFT-2216 on MALT1-mediated BCL10 cleavage in non-GCB DLBCL cell lines. A decrease in cleaved BCL10 and accumulation of uncleaved BCL10 were observed in OCI-Ly3 and RI-1 cells treated with the MALT1 caspase-inhibiting peptide Z-VRPR-FMK (Fig. 4A). In contrast, only uncleaved BCL10 was detected in RC-K8 cells, which lack MALT1 activity (ref. 38; Fig. 4A). These results are consistent with the antiproliferative effect of the low molecular weight MALT1 inhibitor safimaltib in DLBCL tumor cell lines. The IC_50_ values of safimaltib were 1.793, 7.786, and >10 µmol/L for OCI-Ly3, RI-1, and RC-K8 cells, respectively (Supplementary Table S4). FPFT-2216 also induced a decrease in cleaved BCL10 and accumulation of uncleaved BCL10 in OCI-Ly3 and RI-1 cells (Fig. 4A). Lenalidomide and iberdomide did not affect cleaved BCL10 in any cell line (Fig. 4A).

We then examined the effect of CK1α G40N mutant expression on BCL10 cleavage inhibition by FPFT-2216. Z-VRPR-FMK treatment inhibited MALT1-mediated BCL10 cleavage in RI-1 cells regardless of the CK1α G40N mutant (Fig. 4B). A decrease in cleaved BCL10 and accumulation of uncleaved BCL10 were observed in the FPFT-2216–treated parental and control RI-1 cells (Fig. 4B). In contrast, FPFT-2216 did not inhibit MALT1-mediated BCL10 cleavage in the RI-1 expressing CK1α G40N mutant (Fig. 4B), indicating that FPFT-2216 inhibits BCL10 cleavage via CK1α degradation.

Given the reduced expression of phosphorylated IκBα in non-GCB DLBCL cell lines with decreased CK1α expression (19), we evaluated the effect of FPFT-2216 on IκBα phosphorylation. To this end, we used the proteasome inhibitor MG-132 to detect phosphorylated IκBα, which is reportedly degraded rapidly by the proteasome (39) and accumulates to become detectable in a DLBCL cell line treated with MG-132 (40). FPFT-2216 decreased CK1α and A/I protein abundance in a time-dependent manner in OCI-Ly3 cells (Supplementary Fig. S2). Meanwhile, cotreatment with MG-132 for 1 or 6 hours caused accumulation of ubiquitinated proteins and inhibition of the FPFT-2216 effects (Supplementary Fig. S2). Hence, FPFT-2216 likely induces protein degradation via the proteasome pathway. In contrast, compared with Z-138 cells (Fig. 1E), extended treatment (∼24 hours) was required to reduce CK1α in OCI-Ly3 cells (Fig. 4A; Supplementary Fig. S2), likely due to its low ubiquitin/proteasome activity. Hence, to evaluate IκBα phosphorylation under reduced CK1α protein levels, we treated OCI-Ly3 cells with MG-132 following 23 hours of FPFT-2216 treatment. The CRBN neosubstrate-degrading activity of FPFT-2216 and thalidomide derivatives was detected 1 hour posttreatment with MG-132 (Fig. 4C). Furthermore, BAY 11-7082 inhibited IκBα phosphorylation, while FPFT-2216 decreased the amount of phosphorylated IκBα and exhibited stronger activity than thalidomide derivatives (Fig. 4C).

The CK1α/CBM complex/NFκB pathway is also involved in T-cell activation and IL2 production (19, 20). Therefore, we examined whether the inhibitory activity of FPFT-2216 on IκBα phosphorylation could be detected in Jurkat cells, a T-cell leukemia cell line. PMA/ionomycin (P/I)-induced IκBα phosphorylation was suppressed by BAY 11-7082 treatment (Fig. 4D). FPFT-2216 markedly decreased phosphorylated IκBα abundance, whereas thalidomide derivatives did not (Fig. 4D). FPFT-2216 exhibited strong inhibitory activity against P/I-induced NFκB phosphorylation similar to that of BAY 11-7082 (Fig. 4D). Unlike their action on IκBα, thalidomide derivatives inhibited NFκB phosphorylation (Fig. 4D). Finally, we measured the amount of IL2 produced by Jurkat and PBMCs under anti-CD3 antibody stimulation. FPFT-2216 enhanced IL2 production similar to other thalidomide derivatives (Supplementary Table S5; refs. 41, 42).

### FPFT-2216 Exhibits Antitumor Activity in Cell Line-derived Xenograft and PDX Models and Enhances the *In Vivo* Antitumor Activity of the MDM2 Inhibitor and Anti-human CD20 Antibody

**FIGURE 5:**
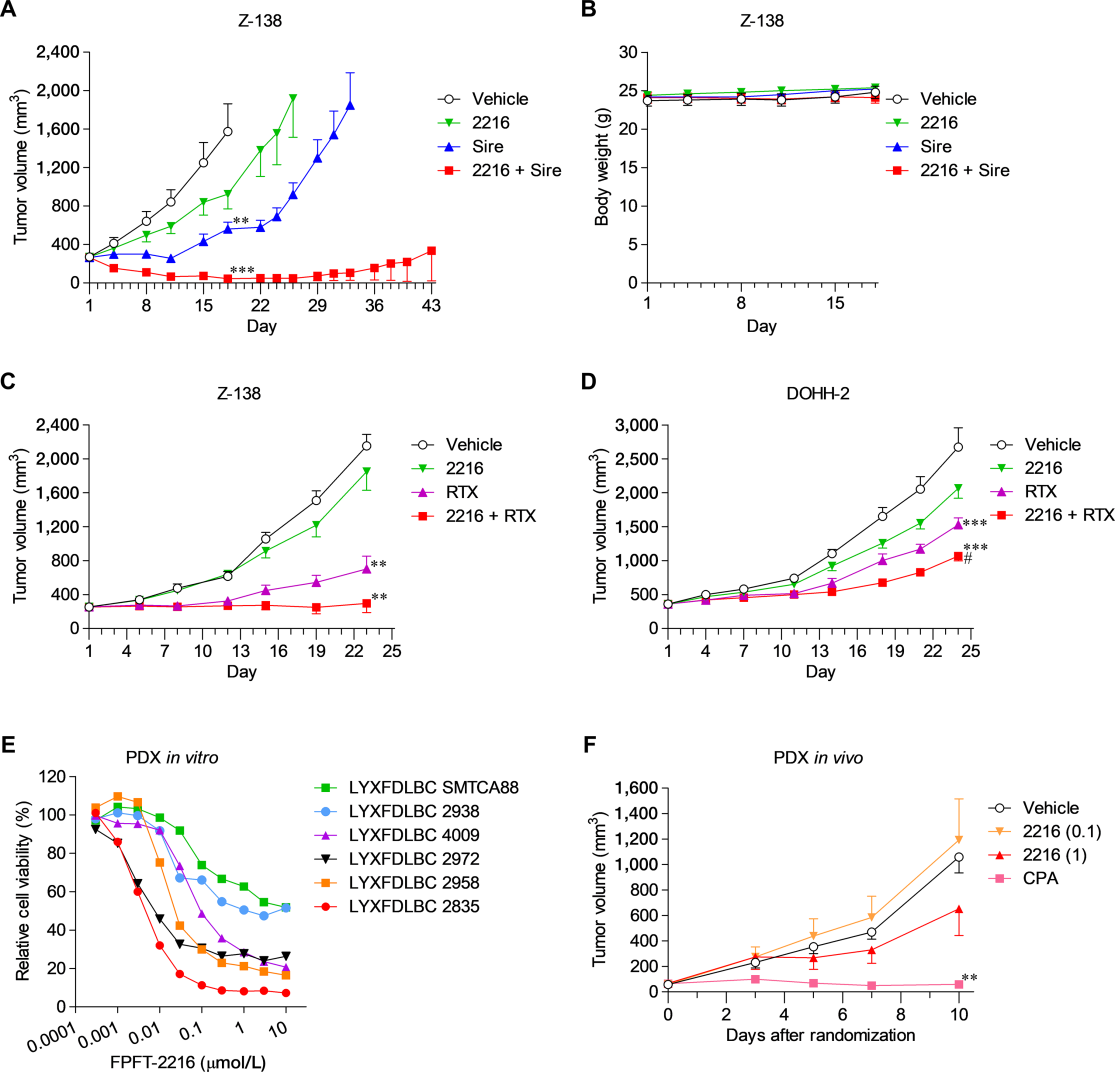
Antitumor activity of FPFT-2216 in CDX and PDX models. FPFT-2216 (10 mg/kg) and siremadlin (100 mg/kg) were administered alone or in combination to mice subcutaneously transplanted with Z-138 cells for three weeks. Tumor volume (**A**) during the subsequent 3-week washout period and body weight (**B**) during the treatment period are shown (mean ± SEM, *n* = 7). **, *P* < 0.01; ***, *P* < 0.001 versus vehicle-treated group (Tukey test). **C,** Tumor volume when FPFT-2216 (1 mg/kg) and rituximab (30 µg/mouse) were administered alone or in combination to mice subcutaneously transplanted with Z-138 cells (mean ± SEM, *n* = 10). **, *P* < 0.01 versus vehicle-treated group (Tukey test). **D,** Tumor volume when FPFT-2216 (0.1 mg/kg) and rituximab (30 µg/mouse) were administered alone or in combination to mice subcutaneously transplanted with DOHH-2 cells (mean ± SEM, *n* = 5–6). ***, *P* < 0.001 versus vehicle-treated group, ^#^*P* < 0.05 versus rituximab-treated group (Dunnett test). **E,** Cell viability (%) in non-GCB DLBCL patient-derived tumor cells treated with various concentrations of FPFT-2216 for 3 days. Results are presented as mean values (duplicate). **F,** Tumor volume after administering FPFT-2216 (0.1, 1 mg/kg) or cyclophosphamide (75 mg/kg) to mice subcutaneously transplanted with LYXFDLBC 2835 tumor cells derived from a patient with non-GCB DLBCL (mean ± SEM, *n* = 6). **, *P* < 0.01 versus vehicle-treated group (Dunnett test). Each figure (A, C, D, F) shows the time-dependent change in tumor volume of each group until the first individual animal was euthanized. 2216, FPFT-2216; Sire, siremadlin; RTX, rituximab; CPA, cyclophosphamide.

FPFT-2216 enhanced the antiproliferative activity of MDM2 inhibitors *in vitro* (Fig. 3E; Supplementary Fig. S1C–S1E) and induced CK1α degradation and p53 activation in Z-138 tumor xenografts (Supplementary Fig. S3). Therefore, we evaluated the antitumor activity of combining FPFT-2216 and siremadlin *in vivo*. In Z-138-transplanted mice, the administration of FPFT-2216 or siremadlin suppressed tumor growth (Fig. 5A; Table 2A). Surprisingly, the FPFT-2216 and siremadlin combination showed a tumor regression effect from day 4 (Fig. 5A; Table 2A). Thus, we performed a follow-up study to assess tumor regrowth after the final FPFT-2216 treatment. The tumor growth delay was measured by determining the number of days required for the tumor volume to reach 2,000 mm^3^ (TTE). The TTE was 34.9 days following treatment with siremadlin alone and 43.0 days after combined siremadlin and FPFT-2216 (*P* < 0.01, Fig. 5A; Table 2A). Tumor regrowth did not occur in 5 of 7 mice in the combination group, suggesting that the TTE, when treated with FPFT-2216, was longer than 43 days. Hence, FPFT-2216 potently enhances the antitumor activity of siremadlin. Neither suppressed body weight gain nor worsened general condition was observed in any treatment group (Fig. 5B).

Given that lenalidomide is used in combination with the anti-human CD20 antibody rituximab to treat certain lymphomas (5), we investigated the antitumor effect of the combinatorial use of FPFT-2216 and rituximab. FPFT-2216 (1 mg/kg) enhanced the antitumor activity of rituximab and relatively suppressed the tumor growth of Z-138 cells (Fig. 5C). The T/C on day 23 was 84.1% in the FPFT-2216 monotherapy, 23.5% in the rituximab monotherapy, and 2.2% in the combination group (Table 2B). FPFT-2216 (0.1 mg/kg) also enhanced the antitumor activity of rituximab in mice transplanted with DOHH-2 cells, an FL-derived cell line (Fig. 5D; Table 2C). However, FPFT-2216 (0.03 mg/kg) did not affect the antitumor activity of rituximab. FPFT-2216, combined with rituximab, was well tolerated, with no effect on performance status or weight development.

Furthermore, we performed *in vitro*/*in vivo* studies using non-GCB DLBCL PDX models to evaluate the anti-lymphoma activity of FPFT-2216 in patient tumors refractory to rituximab-based standard therapy (Supplementary Table. S3). The treatment of PDX cells (p53 wild-type cells) with FPFT-2216 resulted in IC_50_ values ranging from 0.005 to 0.104 µmol/L (Fig. 5E; Table 2D; Supplementary Fig. S4). We then evaluated the antitumor activity of FPFT-2216 in LYXFDLBC 2835-transplanted mice. CPA completely inhibited tumor growth (Fig. 5F). As individuals in the FPFT-2216–treated groups unexpectedly showed aberrant tumor growth and were euthanized on day 10, we compared the tumor volume change on day 10. FPFT-2216 (1 mg/kg) suppressed tumor growth with a T/C of 58.5% (Fig. 5F; Table 2E). However, given that a statistically significant difference in tumor volume was not observed between the vehicle-treated and FPFT-2216–treated groups, FPFT-2216 administration was continued until the animal was euthanized, up to day 21, and TTEs of each animal were recorded. The median TTE was 13.3 days in the vehicle-treated group and 17.8 days in the 1 mg/kg FPFT-2216–treated group, indicating a statistically significant antitumor effect (*P* < 0.05, Table 2E).

## Discussion

**FIGURE 6:**
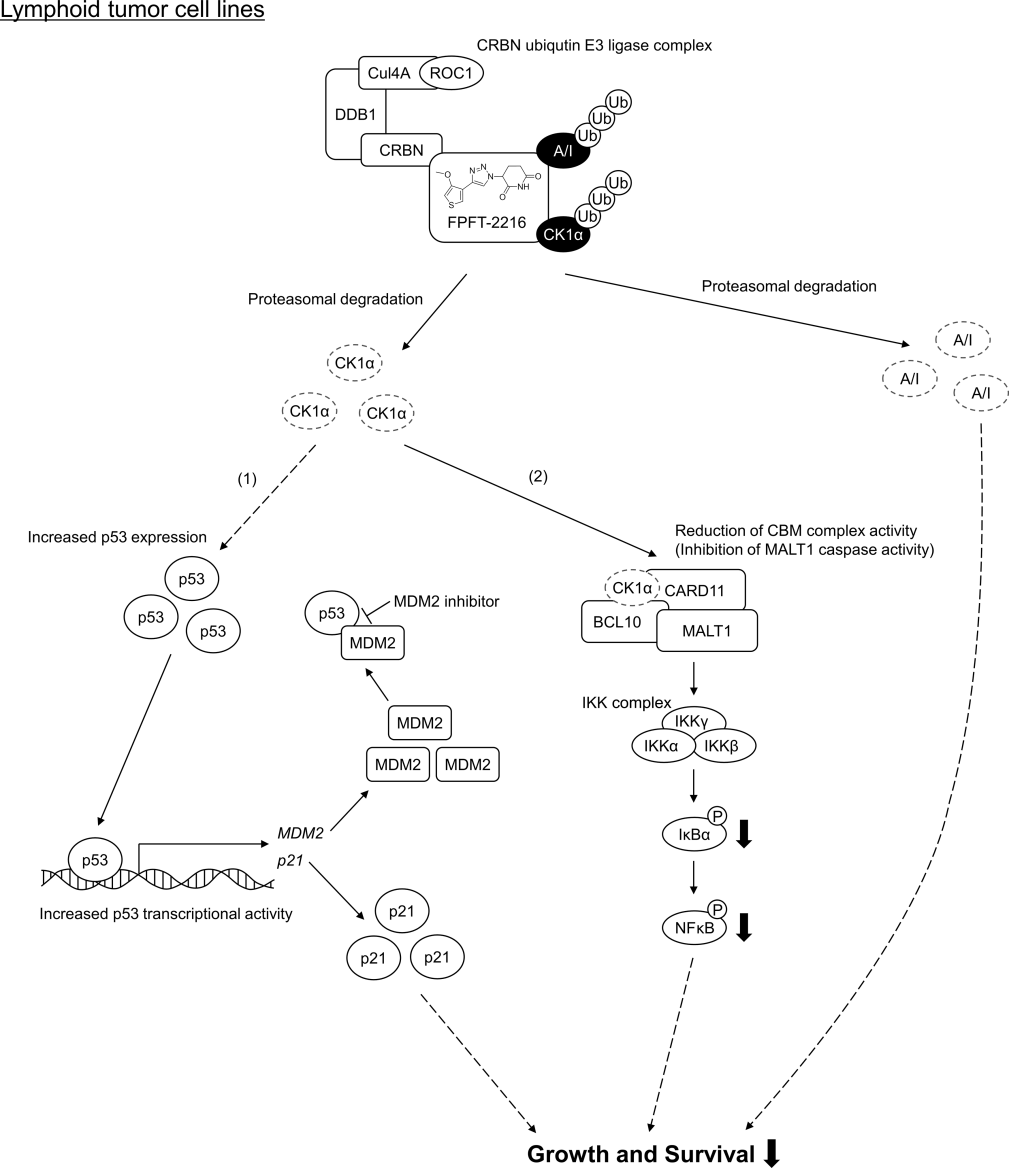
CK1α degradation by FPFT-2216 and subsequent p53 pathway activation and CBM complex/NFκB pathway inhibition. FPFT-2216 enhances CK1α degradation in lymphoid tumor cell lines, resulting in antiproliferative activity via the following two pathways. (1) In the case of p53 wild-type, CK1α degradation activates p53/p21, resulting in cell cycle arrest and growth inhibition. In addition, the binding of MDM2, whose expression is increased by p53, to p53 is suppressed in the presence of an MDM2 inhibitor, further enhancing p53 activation. This is presumed to be the combined action mechanism of FPFT-2216 and an MDM2 inhibitor. (2) CK1α degradation attenuates CBM complex activities, such as MALT1 caspase activity, and suppresses downstream NFκB pathway activation.

The schematic diagram representing the mechanisms underlying the effect of FPFT-2216 via multiple protein degradation is shown in Fig. 6. The anti-lymphoma activity of FPFT-2216 is considered to be mediated by degradation of at least three CRBN neosubstrates, CK1α, and A/I.

Many of the cell lines with growth that was inhibited by FPFT-2216 harbor wild-type p53 (29–33, 35, 43, 44). Therefore, we analyzed the mechanism of action, focusing on the p53 signaling pathway. A previous study reported that CK1α knockdown induces MDM2 and p21 upregulation, which does not occur in p53 knockout cells (45), indicating that CK1α downregulation is directly associated with p53 activation. Consistently, we observed similar results following the treatment of p53 wild-type Z-138 cells with FPFT-2216. Moreover, the p53/p21-upregulating and antiproliferative activities of FPFT-2216 are abolished via exogenous non–CRBN-binding CK1α G40N mutant expression (25–28). Collectively, these results indicate that FPFT-2216 activates the p53 signaling pathway via CK1α degradation and exhibits stronger growth-inhibitory activity than known A/I degraders. Although experiments with the CK1α G40N mutant have been performed previously (25–28), the mechanisms underlying the anti-lymphoma activity and CK1α degradation have not been analyzed; therefore, this study is of great significance in demonstrating the potential of a CK1α-degrading agent as an anticancer drug.

CK1α and p53 are therapeutic targets for del(5q) MDS and AML (13, 17, 34, 46). Lenalidomide is used to treat del(5q) MDS (5), and several CK1α-targeted agents are in clinical trials for AML therapy (4). In our *in vitro* and *in vivo* studies, FPFT-2216 enhanced p53 stabilization and the tumor growth-inhibitory activities of MDM2 inhibitors against hematopoietic malignant cell lines, including AML. In addition, lenalidomide enhances the *in vivo* antitumor activity of siremadlin in Merkel cell carcinoma–derived cell lines (47); hence, FPFT-2216 may prove effective against various cancers by targeting the CK1α/p53 signaling pathway. We are proceeding with combination studies of FPFT-2216 and various signal inhibitors and plan to pursue the cancer-related signals that FPFT-2216 targets.

CK1α degradation by FPFT-2216 was observed *in vitro* regardless of sensitivity to growth inhibition; FPFT-2216 did not exhibit growth inhibition against all p53 wild-type lymphoid tumor cells. That is, FPFT-2216 induced p21 upregulation and potent growth inhibition in OCI-Ly3 not RC-K8 cells. This sensitivity difference might be due to different p53 regulation statuses in positions downstream of CK1α that negative regulator(s) other than MDM2 might act on. For example, C-terminally truncated histone acetyltransferase p300 (p300deltaC) is reportedly expressed in RC-K8 cells (48), while wild-type p300 is expressed in OCI-Ly3 (49). Given that p300deltaC knockdown upregulates p53 expression by 2.4-fold (48), p300deltaC intrinsically suppresses p53 expression in RC-K8 cells. This might partially account for why CK1α degradation had no effect on p21 expression and cell growth in RC-K8 cells.

The CBM complex activates the IκB kinase (IKK) complex by functioning as a scaffold for various proteins and a MALT1 caspase that cleaves multiple substrates, such as BCL10 (50, 51). In non-GCB DLBCL, CK1α-dependent constitutive activation of the NFκB pathway has been observed, implying that the interaction between CK1α and the CBM complex is indispensable in this tumor type (19, 36, 37). In the current study, FPFT-2216 inhibited MALT1-mediated BCL10 cleavage and IκBα phosphorylation in a CK1α-dependent manner, exhibiting stronger effects than that of known A/I degraders. Lenalidomide also inhibits MALT1-mediated BCL10 cleavage and suppresses NFκB activity in non-GCB DLBCL cell lines (22); however, the involvement of CK1α in these activities has not been investigated. Furthermore, the inhibitory activity of FPFT-2216 against the CBM complex/NFκB pathway was also reproduced in the Jurkat T-cell line. In a PMA/ionomycin-treated Jurkat cell line, the thalidomide derivatives did not inhibit IκBα phosphorylation but inhibited NFκB phosphorylation. This finding could be attributed to the inhibition of NFκB phosphorylation through a pathway other than the CBM complex/IKK, such as the Akt signaling pathway (52–54). These results strongly suggest that CK1α-degrading agents potentially act as CBM complex inhibitors (inhibition of MALT1 caspase activity and possibly scaffold function). Given that CBM complex knockdown or IKK inhibition reportedly reduces the viability of the non-GCB DLBCL cell lines (37, 55), the molecular mechanism underlying the action of FPFT-2216 in this study may substantiate its growth-inhibitory activity against non-GCB DLBCL cell lines. CARD11 mutation has been detected in patients with non-GCB DLBCL and the OCI-Ly3 cell line, leading to constitutive NFκB pathway activation (56). Moreover, Z-VRPR-FMK and its analog exhibit stronger inhibitory effects on the proliferation of DLBCL cells harboring a CARD11 mutation, including OCI-Ly3, than non-mutant DLBCL cells (38, 40). This is consistent with our result that, among the three DLBCL cell lines, OCI-Ly3 was the most sensitive to the MALT1 inhibitor safimaltib, followed by RI-1 cells. The CARD11 mutation could render tumors strictly dependent on MALT1 activity; this growth dependency on MALT1 might account for why OCI-Ly3 was the most sensitive to FPFT-2216, and why p53-mutated RI-1 cell growth was inhibited by FPFT-2216. These results collectively support our hypothesis that FPFT-2216 also suppresses the activity of the CBM complex, inhibiting tumor growth in non-GCB DLBCL.

Patients with non-GCB DLBCL have a worse prognosis than those with GCB DLBCL and are less responsive to standard treatments such as R-CHOP (57, 58); thus, new therapeutic agents effective against non-GCB type are eagerly pursued. Exploratory analyses have revealed that the overall response rate of patients with DLBLC treated with lenalidomide is 45.5% for ABC (non-GCB) and 21.4% for GCB (59), while avadomide effectively treats non-GCB DLBCL (60). Meanwhile, FPFT-2216 induced stronger inhibition of non-GCB DLBCL cell growth than lenalidomide or avadomide *in vitro*; moreover, FPFT-2216 suppressed the *in vitro* growth of four of six models of PDX cells from refractory non-GCB DLBCL to standard rituximab-based therapy. Among the four sensitive models mentioned above, the respective cancer driver gene was mutated separately, suggesting that FPFT-2216 could suppress cancer cell growth of heterogeneous origin. However, as experimental results using a small number of patient samples do not necessarily assure drug responses in a large subset of patients, this could be considered a sign of efficacy in patients with non-GCB DLBCL; further efficacy assessments are warranted in future clinical trials. In contrast, the presence of two resistant models, despite both harboring wild-type p53, suggests the existence of resistant mechanisms in patients, similar to those in RC-K8 cells or alternate mechanisms known to occur in CRBN-dependent A/I and CK1α degradation processes. For example, multiple CRBN mutations were found to be associated with acquired resistance to lenalidomide or pomalidomide in multiple myeloma (61). Given the limited number of commercially available PDX models for non-GCB DLBCL suitable for chronic administration of FPFT-2216, only one model, that is, LYXFDLBC 2835, was used for our *in vivo* efficacy study; FPFT-2216 also exhibited antitumor activity *in vivo*. On the basis of these findings, FPFT-2216 is a potential alternative to current therapies against non-GCB DLBCL.

In addition to CK1α, FPFT-2216 potently degrades A/I (11, 23). A/I knockout induces cell death in DLBCL cell lines, and A/I degradation plays a central role in the anti-DLBCL action of avadomide (62). Therefore, A/I degradation contributes, at least in part, to the antiproliferative activity of FPFT-2216 against DLBCL cell lines. In REC-1 (MCL) and Daudi (Burkitt lymphoma) cell lines, FPFT-2216 showed growth-inhibitory activity similar to that of the A/I-degrader iberdomide, suggesting that A/I degradation may also be cytostatic in lymphoma cell lines, such as MCL and BL. As hitherto discussed, FPFT-2216 is considered to behave differently through various oncogenic signaling pathways in individual lymphoma cell lines. Although a clear-cut relationship has not been described between p53 mutation and FPFT-2216 efficacy, each signaling pathway responsible for cell growth inhibition by FPFT-2216 has likely been characterized in this study.

Another aspect of A/I degradation by FPFT-2216 involves the repression of *IL2* by A/I (41). More specifically, IL2 production was enhanced by FPFT-2216 treatment in the Jurkat cell line and anti-CD3 antibody-stimulated PBMCs. Thus, the A/I-degrading activity of FPFT-2216 contributes to its tumor growth-inhibitory and immunomodulatory effects.

As thalidomide derivatives with a glutarimide moiety were inactive in mouse cells (23), the *in vivo* antitumor activity of FPFT-2216 may reflect its direct effect on human tumor cells. Given that we could not evaluate the *in vivo* antitumor activity of FPFT-2216 using tumor-bearing mice with humanized immune cells, we may have underestimated its potential antitumor activity in clinical settings.

We issued a patent application (US11299485B2) covering the invention of FPFT-2216, which has a novel chemical structure comprising a triazole and thiophene ring. Gemechu and colleagues reported that FPFT-2216 exhibits more potent CK1α and A/I degradation activity than lenalidomide (23). Furthermore, FPFT-2216 can degrade PDE6D via CRBN-mediated proteasomal degradation (11).

In conclusion, we discovered that FPFT-2216 suppresses the growth of certain lymphoid tumor cells more than known A/I degraders. This growth-inhibitory effect is mediated by activation of the p53 signaling pathway and inhibition of the CBM complex/NFκB pathway via CK1α degradation. These pharmacologic actions induced by CK1α degradation are speculated to be the mechanism through which FPFT-2216 exhibits superior antitumor activity to that of known A/I degraders. Moreover, *in vivo*, FPFT-2216 elicited a potent tumor regression effect in combination with the MDM2 inhibitor siremadlin and enhanced the antitumor activity of the anti-human CD20 antibody rituximab. Furthermore, FPFT-2216 exhibited inhibitory activity against the *in vitro*/*in vivo* proliferation of non-GCB DLBCL patient-derived tumor cells. These findings suggest that targeting CK1α and A/I with FPFT-2216 is useful for treating hematopoietic malignancies, including lymphoma.

## Supplementary Material

Figure S1 Figure S1 shows that FPFT-2216 activates p53 signaling pathway via CK1α degradation and enhances the anti-proliferative activity of MDM2 inhibitors.

Figure S2 Figure S2 shows that FPFT-2216 degrades CK1α via the proteasome system.

Figure S3 Figure S3 shows the in vivo CK1α-degrading and p53-upregulating activity of FPFT-2216.

Figure S4 Figure S4 shows the genetic characterization of PDX models used in this study.

Table S1 Table S1 is the list of cell lines used in this study.

Table S2 Table S2 is the list of primary antibodies used for western blotting in this study.

Table S3 Table S3 shows patients information on DLBCL PDX models used in this study.

Table S4 Table S4 shows the anti-proliferative effect of MALT1 inhibitor safimaltib in lymphoid tumor cell lines.

Table S5 Table S5 shows the enhancing effect of FPFT-2216 on IL-2 production.
