# Supplementary material for: FPFT-2216, a Novel Anti-lymphoma Compound, Induces Simultaneous Degradation of IKZF1/3 and CK1α to Activate p53 and Inhibit NFκB Signaling
Source: Cancer Res Commun. 2024 Feb 6;4(2):312–27. doi: 10.1158/2767-9764.CRC-23-0264 (PMC10846380; doi:10.1158/2767-9764.CRC-23-0264)
Supplement: Figure S1 — shows that FPFT-2216 activates p53 signaling pathway via CK1α degradation and enhances the anti-proliferative activity of MDM2 inhibitors. [file crc-23-0264-s01.pdf]

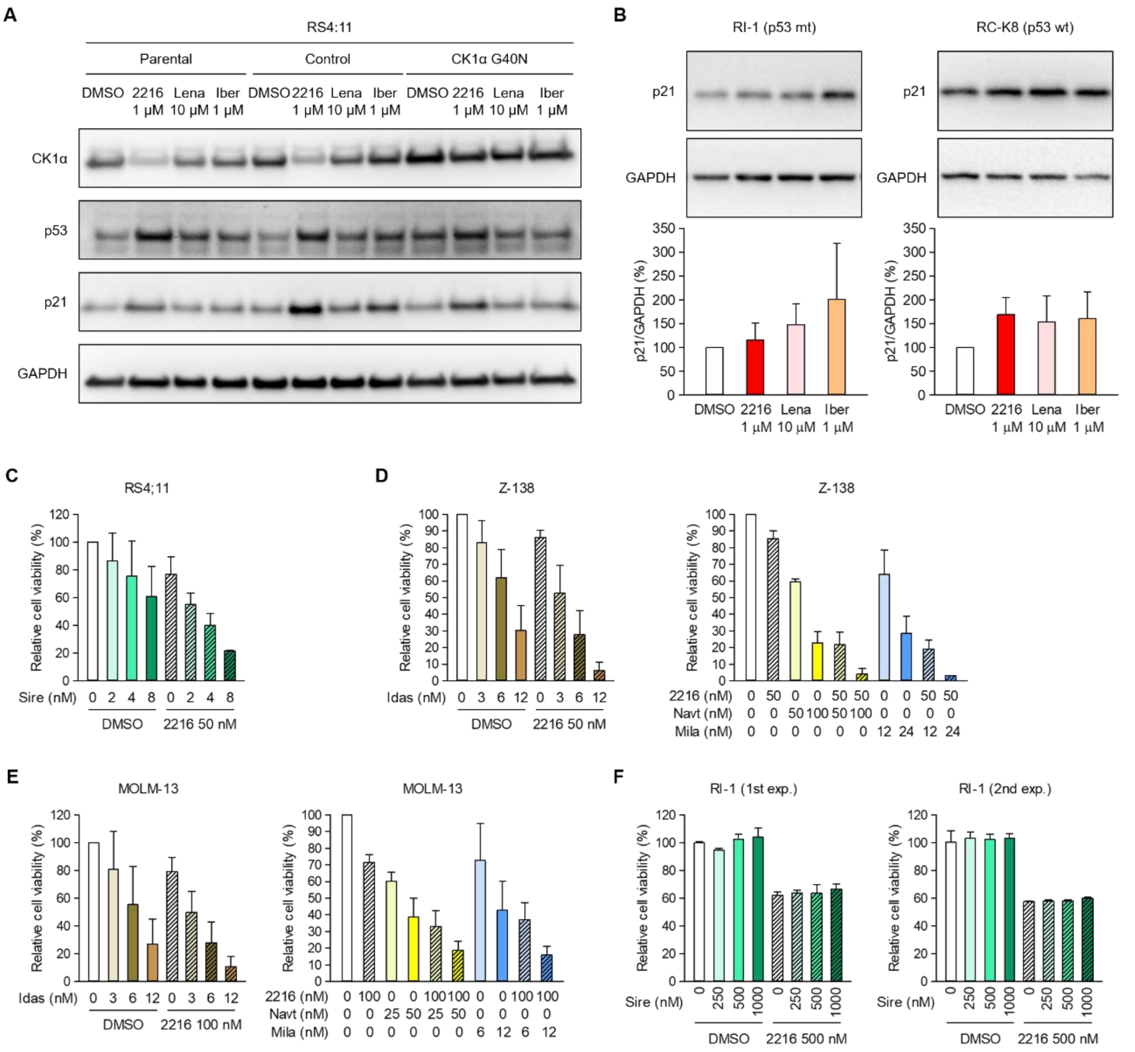


**Supplementary Figure S1.** Activation of the p53 signaling pathway via CK1α degradation by FPFT-2216 and its potentiating effect on the anti-proliferative activity of MDM2 inhibitors. **A**: Western blot analysis of RS4;11 (parental), green fluorescent protein-expressing RS4;11 (control), or CK1α G40N mutant-expressing RS4;11 cells cultured for 6 h in the presence of various compounds at different concentrations. **B**: Western blot analysis of p21 protein in RI-1 and RC-K8 cells cultured for 24 h in the presence of various compounds at different concentrations. The p21 band intensity was normalized to GAPDH, and the p21/GAPDH band intensity ratio (%) for each compound treatment was determined with the p21/GAPDH band intensity ratio for DMSO-treated cells taken as 100% (mean ± SD, n = 3). Representative results from two (**A**) or three (**B**) independent experiments are shown. GAPDH was used as a loading control. **C**–**F**: Cell viability (%) of RS4;11, Z-138, MOLM-13, and RI-1 cells cultured for three days in the presence of FPFT-2216 and MDM2 inhibitors (siremadlin, idasanutlin, navtemadlin, and

milademetan) at the concentrations shown in the figure. Results are shown as the mean ± SD (n = 3) (**C–E**). Two independent experiments were performed, each in triplicate. Results are shown as the mean ± SD (n = 3) (**F**).

2216, FPFT-2216; Lena, lenalidomide; Iber, iberdomide; Sire, siremadlin; Idas, idasanutlin; Navt, navtemadlin; Mila, milademetan.
