# Supplementary material for: FPFT-2216, a Novel Anti-lymphoma Compound, Induces Simultaneous Degradation of IKZF1/3 and CK1α to Activate p53 and Inhibit NFκB Signaling
Source: Cancer Res Commun. 2024 Feb 6;4(2):312–27. doi: 10.1158/2767-9764.CRC-23-0264 (PMC10846380; doi:10.1158/2767-9764.CRC-23-0264)
Supplement: Figure S2 — shows that FPFT-2216 degrades CK1α via the proteasome system. [file crc-23-0264-s02.pdf]

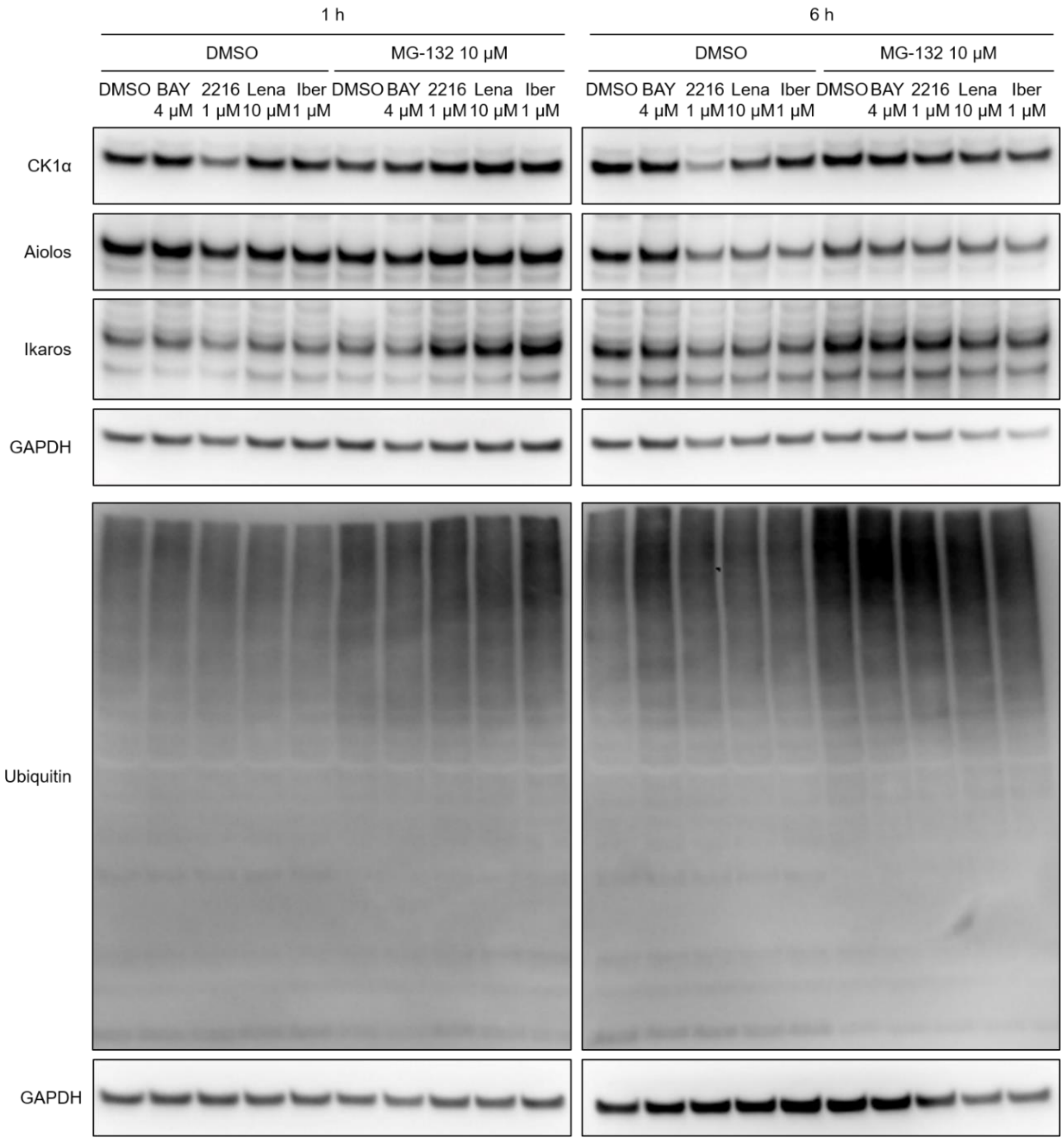

Supplementary Figure S2. CRBN neosubstrate degradation via proteasome induced by FPFT-2216. Western blot analysis of OCI-Ly3 cells cultured for 1 or 6 h in the presence of compounds at different concentrations. Representative results from two independent experiments are shown. GAPDH was used as a loading control.

BAY, BAY 11-7082; 2216, FPFT-2216; Lena, lenalidomide; Iber, iberdomide.
