# Supplementary material for: FPFT-2216, a Novel Anti-lymphoma Compound, Induces Simultaneous Degradation of IKZF1/3 and CK1α to Activate p53 and Inhibit NFκB Signaling
Source: Cancer Res Commun. 2024 Feb 6;4(2):312–27. doi: 10.1158/2767-9764.CRC-23-0264 (PMC10846380; doi:10.1158/2767-9764.CRC-23-0264)
Supplement: Figure S3 — shows the in vivo CK1α-degrading and p53-upregulating activity of FPFT-2216. [file crc-23-0264-s03.pdf]

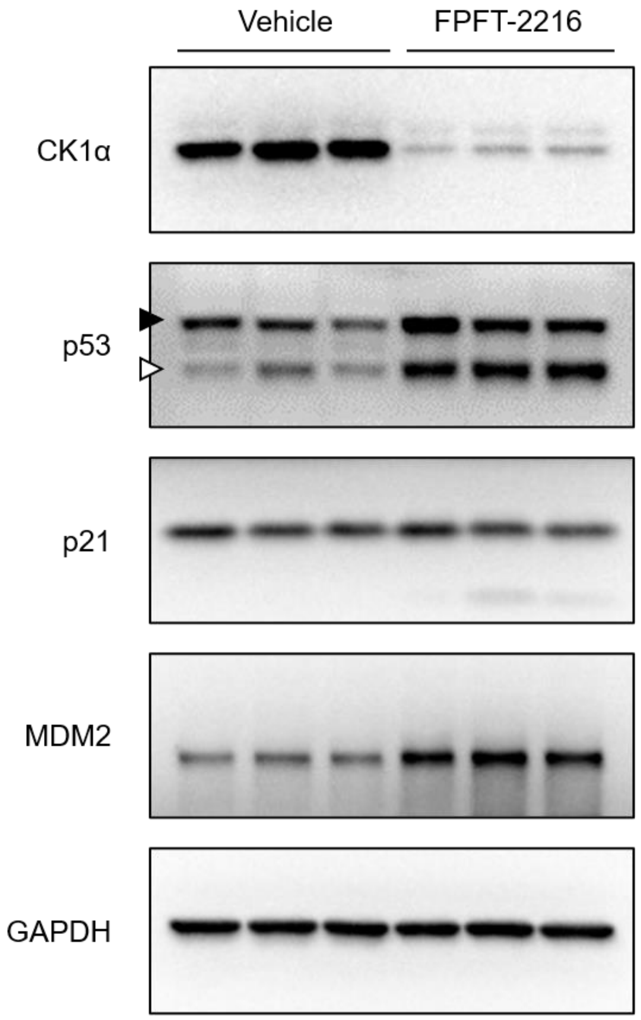

Supplementary Figure S3. CK1α degradation and p53 activation induced by FPFT-2216 *in vivo*. Z-138 tumor-bearing mice were administered FPFT-2216 (10 mg/kg) once daily for four days, and tumors were harvested 6 h after the 4th administration (n = 3). CK1α, p53, p21, MDM2, and GAPDH protein abundances were measured in the tumor lysates by western blot analysis. Closed triangle: p53 (53 kDa), open triangle: p53 isoform (approximately 40 kDa). GAPDH was used as a loading control.
