# Supplementary material for: FPFT-2216, a Novel Anti-lymphoma Compound, Induces Simultaneous Degradation of IKZF1/3 and CK1α to Activate p53 and Inhibit NFκB Signaling
Source: Cancer Res Commun. 2024 Feb 6;4(2):312–27. doi: 10.1158/2767-9764.CRC-23-0264 (PMC10846380; doi:10.1158/2767-9764.CRC-23-0264)
Supplement: Figure S4 — shows the genetic characterization of PDX models used in this study. [file crc-23-0264-s04.pdf]

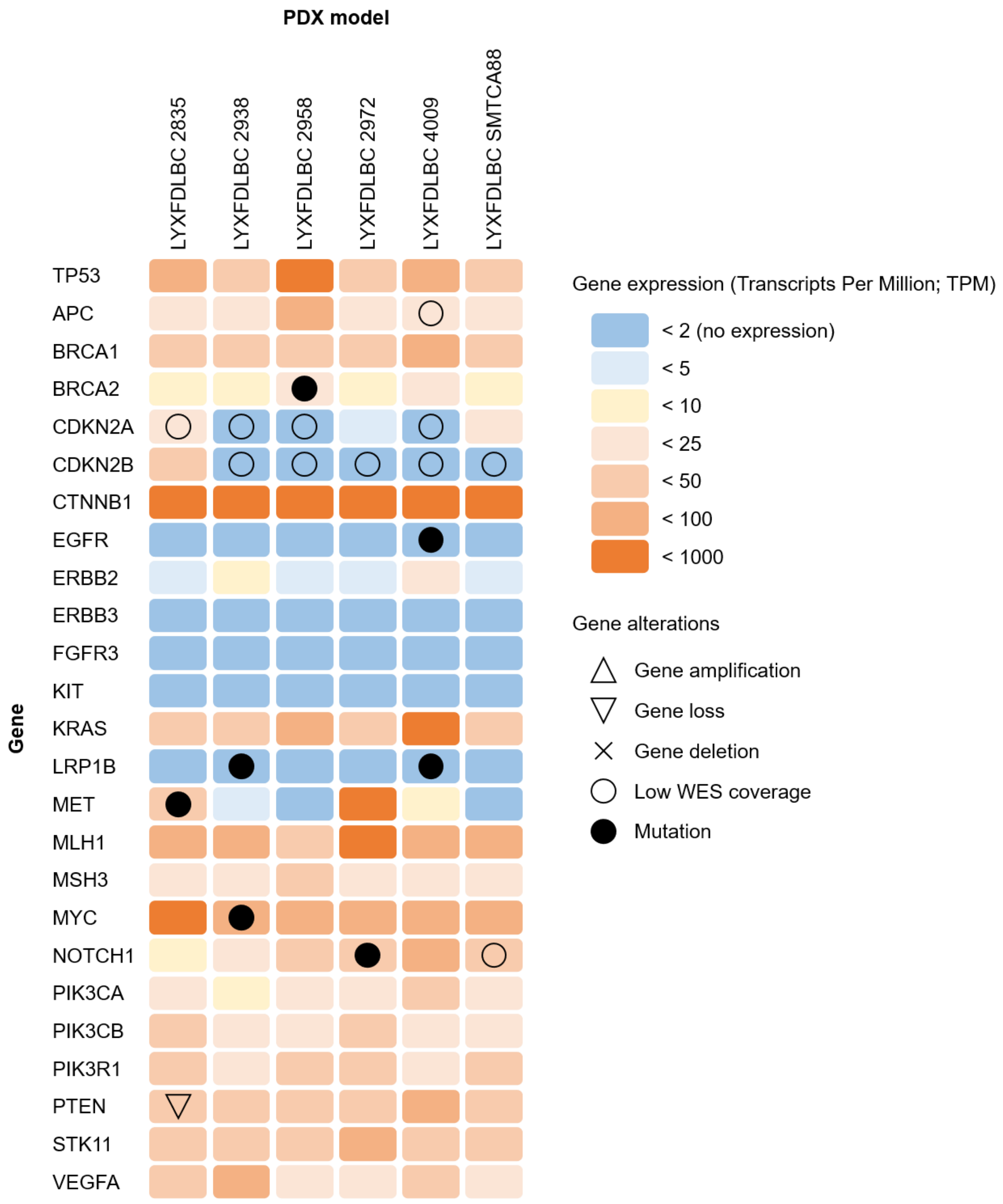

Supplementary Figure S4. Gene alteration landscape of PDX models. The RNA-seq and WES data of PDX models used in this study were obtained from the Charles River Tumor Model Compendium (https://compendium.criver.com/). This image has been modified from its original version.
