# Supplementary material for: FPFT-2216, a Novel Anti-lymphoma Compound, Induces Simultaneous Degradation of IKZF1/3 and CK1α to Activate p53 and Inhibit NFκB Signaling
Source: Cancer Res Commun. 2024 Feb 6;4(2):312–27. doi: 10.1158/2767-9764.CRC-23-0264 (PMC10846380; doi:10.1158/2767-9764.CRC-23-0264)
Supplement: Table S1 — is the list of cell lines used in this study. [file crc-23-0264-s05.pdf]

Supplementary Table S1. Cell lines used in this study

Tumor type	Cell line	Source	Year of purchase	Culture medium
DLBCL (GCB)	WSU-DLCL2 (RRID:CVCL_1902)	DSMZ	2018	10% FBS/RPMI 1640
	RC-K8 (RRID:CVCL_1883)	JCRB	2021	20% FBS/RPMI 1640
	SU-DHL-4 (RRID:CVCL_0539)	ATCC	2016	10% FBS/RPMI 1640
	Pfeiffer (RRID:CVCL_3326)	ATCC	2016	10% FBS/RPMI 1640
DLBCL (non-GCB)	U-2932 (RRID:CVCL_1896)	DSMZ	2018	10% FBS/RPMI 1640
	RI-1 (RIVA) (RRID:CVCL_1885)	DSMZ	2018	10% FBS/RPMI 1640
	OCI-Ly3 (RRID:CVCL_8800)	DSMZ	2021	20% FBS/RPMI 1640
	SU-DHL-2 (RRID:CVCL_9550)	ATCC	2016	10% FBS/RPMI 1640
MCL	MAVER-1 (RRID:CVCL_1831)	ATCC	2018	10% FBS/RPMI 1640
	Z-138 (RRID:CVCL_B077)	ATCC	2018	10% FBS/RPMI 1640
	GRANTA-519 (RRID:CVCL_1818)	DSMZ	2020	10% FBS/DMEM
	JVM-2 (RRID:CVCL_1319)	DSMZ	2020	10% FBS/RPMI 1640
	MINO (RRID:CVCL_1872)	DSMZ	2020	20% FBS/RPMI 1640
	JeKo-1 (RRID:CVCL_1865)	ATCC	2018	20% FBS/RPMI 1640
	REC-1 (RRID:CVCL_1884)	ATCC	2018	10% FBS/RPMI 1640
FL	RL (RRID:CVCL_1660)	DSMZ	2020	20% FBS/RPMI 1640
	DOHH-2 (RRID:CVCL_1179)	DSMZ	2018	10% FBS/RPMI 1640
	Minami-1 (RRID:CVCL_8528)	JCRB	2018	10% FBS/RPMI 1640
BL	CA46 (RRID:CVCL_1101)	ATCC	2018	20% FBS/RPMI 1640
	Daudi (RRID:CVCL_0008)	JCRB	2009	10% FBS/RPMI 1640
	Ramos(RA1) (RRID:CVCL_0597)	JCRB	2018	10% FBS/RPMI 1640
ALL	SEM (RRID:CVCL_0095)	DSMZ	2018	10% FBS/RPMI 1640
	NALM-6 (RRID:CVCL_0092)	DSMZ	2018	10% FBS/RPMI 1640
	Kasumi-8 (RRID:CVCL_8487)	JCRB	2018	10% FBS/RPMI 1640
	KHM-2B (RRID:CVCL_8526)	JCRB	2018	10% FBS/RPMI 1640
	REH (RRID:CVCL_1650)	DSMZ	2018	10% FBS/RPMI 1640
	RS4;11 (RRID:CVCL_0093)	DSMZ	2018	10% FBS/RPMI 1640
	Kasumi-7 (RRID:CVCL_8486)	JCRB	2018	20% FBS/RPMI 1640
	Kasumi-10 (RRID:CVCL_8485)	JCRB	2018	20% FBS/RPMI 1640
AML	MOLM-13 (RRID:CVCL_2119)	DSMZ	2018	10% FBS/RPMI 1640
T-ALL	Jurkat (RRID:CVCL_0065)	ATCC	2009	10% FBS/RPMI 1640
Normal	HEK293T (RRID:CVCL_0063)	Horizon Discovery	2016	10% FBS/DMEM
	PBMCs	Healthy volunteers	-	10% FBS/RPMI 1640

Abbreviations: DLBCL, diffuse large B-cell lymphoma; GCB, germinal center B-cell; MCL, mantle cell lymphoma; FL, follicular lymphoma; BL, Burkitt lymphoma; ALL, acute lymphoblastic leukemia; AML, acute myeloid leukemia; T-ALL, acute T-cell leukemia; PBMCs, peripheral blood mononuclear cells; JCRB, Japanese Collection of Research Bioresources.
