# Supplementary material for: FPFT-2216, a Novel Anti-lymphoma Compound, Induces Simultaneous Degradation of IKZF1/3 and CK1α to Activate p53 and Inhibit NFκB Signaling
Source: Cancer Res Commun. 2024 Feb 6;4(2):312–27. doi: 10.1158/2767-9764.CRC-23-0264 (PMC10846380; doi:10.1158/2767-9764.CRC-23-0264)
Supplement: Table S2 — is the list of primary antibodies used for western blotting in this study. [file crc-23-0264-s06.pdf]

Supplementary Table S2. Primary antibodies used in this study for western blotting

Antibody target	Supplier	Catalog number	RRID	Dilution
Aiolos	Cell Signaling Technology	15103	AB_2744524	1:1000
Ikaros		14859	AB_2744523	1:1000
p21		2947	AB_823586	1:1000
MDM2		86934	AB_2784534	1:1000
BCL10		4237	AB_2228005	1:1000
Phospho-IκBα		9246	AB_2267145	1:1000
IκBα		4814	AB_390781	1:1000
Phospho-NF-κB		3033	AB_331284	1:1000
NF-κB		8242	AB_10859369	1:1000
Ubiquitin		3636	AB_331292	1:1000
alpha tubulin	Abcam	ab176560	AB_2860019	1:1000
CK1α		ab206652	AB_2925161	1:1000
GAPDH		ab8245	AB_2107448	1:10,000–50,000
GAPDH		ab128915	AB_11143050	1:10,000
GAPDH		ab201822	AB_2927782	1:10,000 or 50,000
p53	Santa Cruz Biotechnology	sc-126	AB_628082	1:500 or 1000
PDE6D	Atlas Antibodies	HPA037433	AB_2675475	0.4 µg/mL[a]

[a]Working concentration
