# Supplementary material for: FPFT-2216, a Novel Anti-lymphoma Compound, Induces Simultaneous Degradation of IKZF1/3 and CK1α to Activate p53 and Inhibit NFκB Signaling
Source: Cancer Res Commun. 2024 Feb 6;4(2):312–27. doi: 10.1158/2767-9764.CRC-23-0264 (PMC10846380; doi:10.1158/2767-9764.CRC-23-0264)
Supplement: Table S3 — shows patients information on DLBCL PDX models used in this study. [file crc-23-0264-s07.pdf]

Supplementary Table S3. Non-GCB DLBCL PDX models used in this study

PDX model	Sex	Age	Site of origin	Pre-implantation chemotherapy
LYXFDLBC 2835	Male	72	CNS	6 cycles R-CHOP + 2 cycles R-MTX
LYXFDLBC 2938	Male	57	Brain	6 cycles R-CHOP + 2 cycles R-MTX
LYXFDLBC 2958	Female	61	Brain	6 cycles R-CHOP + 3 cycles R-ICE + BEAM + autologous stem cell transplant
LYXFDLBC 2972	Male	70	Bone marrow	R-CHOP + R-MTX
LYXFDLBC 4009	Female	68	Lymph node	8 cycles R-CHOP + 2 cycles R-DHAOx + 2 cycles R-GEMOX + 2 cycles EPOCH-R + R-VIM + 2 cycles R-CBVPP + 2 cycles R-PEBEN
LYXFDLBC SMTCA88	Not known	Not known	Ascites	Not known

Abbreviations: GCB, germinal center B-cell; DLBCL, diffuse large B-cell lymphoma; CNS, central nervous system; R, rituximab; CHOP, cyclophosphamide + doxorubicin + vincristine + prednisone; MTX, methotrexate; ICE, ifosfamide + carboplatin + etoposide; BEAM, carmustine + etoposide + cytarabine + melphalan; DHAOx, dexamethasone + high dose cytarabine + oxaliplatin; GEMOX, gemcitabine + oxaliplatin; EPOCH, etoposide + prednisone + vincristine + cyclophosphamide + doxorubicin; VIM, etoposide + ifosfamide + methotrexate; CBVPP, cyclophosphamide + carmustine + vinblastine + procarbazine + prednisone; PEBEN, pixantrone + etoposide + bendamustine.
