# Supplementary material for: FPFT-2216, a Novel Anti-lymphoma Compound, Induces Simultaneous Degradation of IKZF1/3 and CK1α to Activate p53 and Inhibit NFκB Signaling
Source: Cancer Res Commun. 2024 Feb 6;4(2):312–27. doi: 10.1158/2767-9764.CRC-23-0264 (PMC10846380; doi:10.1158/2767-9764.CRC-23-0264)
Supplement: Table S4 — shows the anti-proliferative effect of MALT1 inhibitor safimaltib in lymphoid tumor cell lines. [file crc-23-0264-s08.pdf]

Supplementary Table S4. IC_{50} of safimaltib on lymphoid tumor cell line proliferation

Cell line	IC_{50} (μM)
OCI-Ly3	1.793
RI-1	7.786
RC-K8	> 10
Z-138	> 10
RS4;11	> 10

Lymphoid tumor cell lines treated with safimaltib at 0.01–10 μM were seeded in 96-well plates and cultured at 37°C under 5% CO_2 for three days. A WST-8 kit (Kishida Chemical Laboratory) was used to evaluate cell viability. The percentage of absorbance in the safimaltib-treated cells was calculated as a value relative to the absorbance in DMSO-treated cells (control), designated as 100%, and defined as the cell viability (%). The IC_{50} value was calculated with Graph Pad Prism 5.04 (GraphPad Software) using the cell viability results of three independent experiments.
