# Supplementary material for: FPFT-2216, a Novel Anti-lymphoma Compound, Induces Simultaneous Degradation of IKZF1/3 and CK1α to Activate p53 and Inhibit NFκB Signaling
Source: Cancer Res Commun. 2024 Feb 6;4(2):312–27. doi: 10.1158/2767-9764.CRC-23-0264 (PMC10846380; doi:10.1158/2767-9764.CRC-23-0264)
Supplement: Table S5 — shows the enhancing effect of FPFT-2216 on IL-2 production. [file crc-23-0264-s09.pdf]

Supplementary Table S5. Enhancing effect of FPFT-2216 on IL-2 production

	EC_{150} (nM)	
	Jurkat cells	PBMCs
FPFT-2216	90.5	0.2
Lenalidomide	157.3	0.5
Pomalidomide	14.7	0.1
Iberdomide	N. D.	0.01

Under anti-CD3 antibody stimulation, various compounds were added to Jurkat cells at 0.001–100 μM and PBMCs at 0.001 nM–100 μM. After 48 h of culture, the amount of IL-2 in the culture supernatant was quantitated by ELISA. Considering the amount of IL-2 produced with DMSO treatment as 100%, the relative value (%) with each compound treatment was calculated to determine 150% effective concentration (EC_{150}). The EC_{150} indicates the compound concentration where the relative IL-2 production rate reaches 150% (two trials for Jurkat cells and five for PBMCs). Analysis was performed using Graph Pad Prism 5.04 (GraphPad Software).

PBMCs, peripheral blood mononuclear cells; N. D., Not determined.
